# Gastroesophageal circulating tumor cell crosstalk with peripheral immune system guides CTC survival and proliferation

**DOI:** 10.1038/s41419-025-07530-2

**Published:** 2025-03-29

**Authors:** Tania Rossi, Martina Valgiusti, Maurizio Puccetti, Giacomo Miserocchi, Michele Zanoni, Davide Angeli, Chiara Arienti, Ilaria Pace, Cristian Bassi, Ivan Vannini, Mattia Melloni, Erika Bandini, Milena Urbini, Massimo Negrini, Massimiliano Bonafè, Manuela Ferracin, Giulia Gallerani

**Affiliations:** 1 Biosciences Laboratory, IRCCS Istituto Romagnolo per lo Studio dei Tumori (IRST) “Dino Amadori”, Meldola, Italy; 2 Department of Medical Oncology, IRCCS Istituto Romagnolo per lo Studio dei Tumori (IRST) “Dino Amadori”, Meldola, Italy; 3 Azienda Unità Sanitaria Locale di Imola, Imola, Italy; 4 Preclinic and Osteoncology Unit, Biosciences Laboratory, IRCCS Istituto Romagnolo per lo Studio dei Tumori (IRST) “DinoAmadori”, Meldola, Italy; 5 Unit of Biostatistics and Clinical Trials, IRCCS Istituto Romagnolo per lo Studio dei Tumori (IRST) “Dino Amadori”, Meldola, Italy; 6 Immuno-Gene Therapy Factory, IRCCS Istituto Romagnolo per lo Studio dei Tumori (IRST) “Dino Amadori”, Meldola, Italy; 7 Department of Medical and Surgical Sciences (DIMEC), University of Bologna, Bologna, Italy; 8 Department of Translational Medicine, Laboratorio per le Tecnologie delle Terapie Avanzate (LTTA) Centre, University of Ferrara, Ferrara, Italy; 9 Pathology Unit, Morgagni-Pierantoni Hospital, AUSL Romagna, Forlì, Italy; 10 Department of Translational Medicine, University of Ferrara, Ferrara, Italy; 11 IRCCS Azienda Ospedaliero-Universitaria di Bologna, Bologna, Italy

**Keywords:** Cancer models, Oesophageal cancer, Tumour biomarkers

## Abstract

Tumor dissemination is a key event in tumor progression. During this event, a main role is played by circulating tumor cells (CTCs), immune cells, and their interaction. How the immune system supports the survival and proliferation of CTCs is not fully elucidated. In this study we established an in-vitro co-culture system consisting of immune cells and CTCs from the same patient, which increased the success rate in the establishment of CTC-derived long-term cell cultures. In this system, we characterized the immune cells of successful co-cultures and the signals they exchange with cancer cells, including cytokines and extracellular vesicle (EV) content. Using this protocol, we stabilized four CTC-derived cell lines from patients with metastatic gastroesophageal cancer, which were cultured for over a year and characterized from a genetic and molecular point of view. The four cell lines harbor shared chromosomal aberrations including the amplification at 8q24.21 containing *MYC* and deletion 9p21.3 containing *CDKN2A/B* and the IFN type I cluster. The transcriptomic profile of CTC cell lines is distinct from primary tumors, and we detected the activation of E2F, G2M and MYC pathways and the downregulation of interferon response pathway. Each cell line shows a degree of invasiveness in zebrafish in-vivo, and the most invasive ones share the same mutation in *RAB14* gene. In addition, the four cell lines secrete cell-line specific EVs containing microRNAs that target YAP, BRG1-AKT1, TCF8-HDAC pathways. Overall, we highlight how the immune system plays a key role in the proliferation of CTCs through EV signaling, and how CTC cell line genomic and transcriptomic alterations make these cells less visible from the immune system and likely responsible for the survival advantage in sites distant from the microenvironment of origin.

## Introduction

Gastric, gastroesophageal junction (GEJ), and esophageal cancers often present as a metastatic disease. Their incidence has been rising in the past decades and contribute to 6% of all cancer related deaths [1]. Although many treatment options are available, the prognosis of advanced gastroesophageal cancer (GEC) remains poor [1]. In recent years, the molecular and genomic characteristics of this tumor have been extensively described [2–4], although providing little information about the biological mechanisms of GEC aggressiveness.

Circulating tumor cells (CTCs) are considered key elements for disease progression, considering their pivotal role in the metastatic spread. CTCs are the fraction of a solid tumor that detaches from the primary site and travel within the bloodstream allowing the cancer to spread to distant organs [5, 6]. While numerous CTCs and CTC clusters are shed during the primary tumor growth [7–9] the dissemination process remains highly inefficient, with only a small fraction (approximately 0.02%) of these cells successfully completing the metastatic cascade and forming secondary tumors [10–12].

The number of CTC in the circulation is a prognostic biomarker in many tumor types, and CTC counting in GEC has been recognized to have a prognostic value [13, 14]. In this context, we previously demonstrated that esophageal cancers shed not only epithelial CTCs, but also mesenchymal and hybrid “unconventional” CTCs [15] during treatment, thus confirming the relevance of epithelial to mesenchymal transition (EMT) program activation in cancer evolution [16]. We also described that esophageal CTCs harbor extensive genomic alterations, especially in relapsed patients, enriched for regions associated to the innate immune system pathways [16], but how these aberrations impact on metastasis formation is still an open question.

While CTC count is reaching its full potential in the clinic [17], we still have a limited knowledge about CTC biological role in cancer metastasization. Growing evidence suggests the existence of an active crosstalk between CTCs and non-neoplastic cells [18] including platelets [19–21], cancer-associated fibroblasts CAFs [22–24] and immune cells [25, 26]. Indeed, the cooperation between CTCs and different cell types in the bloodstream influences various aspects of the metastatic cascade [27]. CTC immune escaping is related to their interaction with “educated” platelets [28, 29] and natural killer cells (NK) [30]; while extravasation is promoted by neutrophil assistance [26]. Seminal studies have shown that myeloid-derived suppressor cells (MDSCs) [25] and CAFs [23] are also involved in the metastatic cascade of CTCs outside the primary site by promoting their dissemination and facilitating their metastatic growth.

Understanding the crosstalk between CTCs and non-tumor cells that sustain a favorable metastatic niche and establishing in vitro models of pre-metastatic niche formation is therefore crucial.

In this study, we developed a custom co-culture system for the autologous co-culture of patients derived CTCs and peripheral blood mononuclear cells (PBMCs) to increase the success rate in long-term CTC cell culture establishment and identify the interactions between CTCs and immune cells that are responsible for CTC growth and survival. We found that fibroblasts and monocyte-derived macrophages lead CTC proliferation through a crosstalk consisting of cytokines and extracellular vesicle (EVs) delivered microRNAs (miRNAs).

We propagated four CTC cell lines growing as tumoroids, which recapitulate the pathological characteristics of the original tumor, and investigated their invasive potential in a zebrafish model. We further characterized the CTC specific molecular and genetic features and the content of CTC-derived EVs.

Our study provides convincing evidence that the proliferation of CTCs is supported by immune system cells, possibly through EV content and cytokine crosstalk. As a result of this study, we immortalized four CTC-derived cell lines allowing a step forward to the knowledge of the metastatic cascade of gastroesophageal cancer.

## Results

### Successfully establishment of CTC-derived cell lines from patients with gastroesophageal cancer

After an initial failure in CTC culturing from 18 metastatic GEC patients using approaches based on mono-cell culture (enriched CTCs) [31], we decided to test the hypothesis that CTCs could acquire their full growth and metastatic potential from the interaction with other cells, and specifically immune cells. We settled a co-culture system for CTC propagation using both enriched CTCs and peripheral blood cells from 6 patients with metastatic GEC.

The co-culture system consisted of a well insert comprising Alvetex scaffold technology -a porous polystyrene 3D scaffold [32], in an ultra-low attachment well plate.

The 3D scaffold is seeded with PBMC from patient and the bottom of the well was seeded with CTCs enriched from the same patient. The co-culture was then maintained under serum-free growth conditions with 4% O2.

After 20–30 days, cell aggregates were visible only in the bioreactors. After 25–30 days, growing CTCs were present in four out of six co-cultures and were further propagated outside the bioreactor. All bioreactor components (cells, supernatant, EVs) were collected for subsequent analyses.

Long-term continuous CTC cell lines were established from four patients (LLCTC, NM9CTC, RGCTC, CACTC) and successfully propagated beyond the 20th passage. This result indicates that the bioreactor was successful in 66% of cases, which compared to the 0% success rate of the mono-cell cultured mode is impressive. The clinical and pathological characteristics of the source patients are listed in Table 1. LLCTC and NM9CTC were both derived from esophageal adenocarcinoma. RGCTC and CACTC cell lines were derived from squamous esophageal and gastric cancer, respectively.

**Table 1:** Clinical and pathological characteristics of the source patients of CTC-derived cell lines.

Cell Lines ID/Patient	Primary tumor histology	Grade	Mestatic at diagnosis	Resection of primary tumor	Distant metastasis
LLCTC/4	Esophageal Adenocarcinoma	3	Yes	No	Lymph nodes, Liver, Carcinosis, Adrenal gland
CACTC/14	Diffuse Gastric Cancer	3	Yes	No	Lymph nodes, Adrenal gland
RGCTC/16	Squamous Esophageal Carcinoma	3	Yes	No	Lymph nodes, Carcinosis
NM9CTC/17	Esophageal Adenocarcinoma	ND	No	Yes	Liver, Carcinomatosis, Andrenal gland

**Fig. 1:**
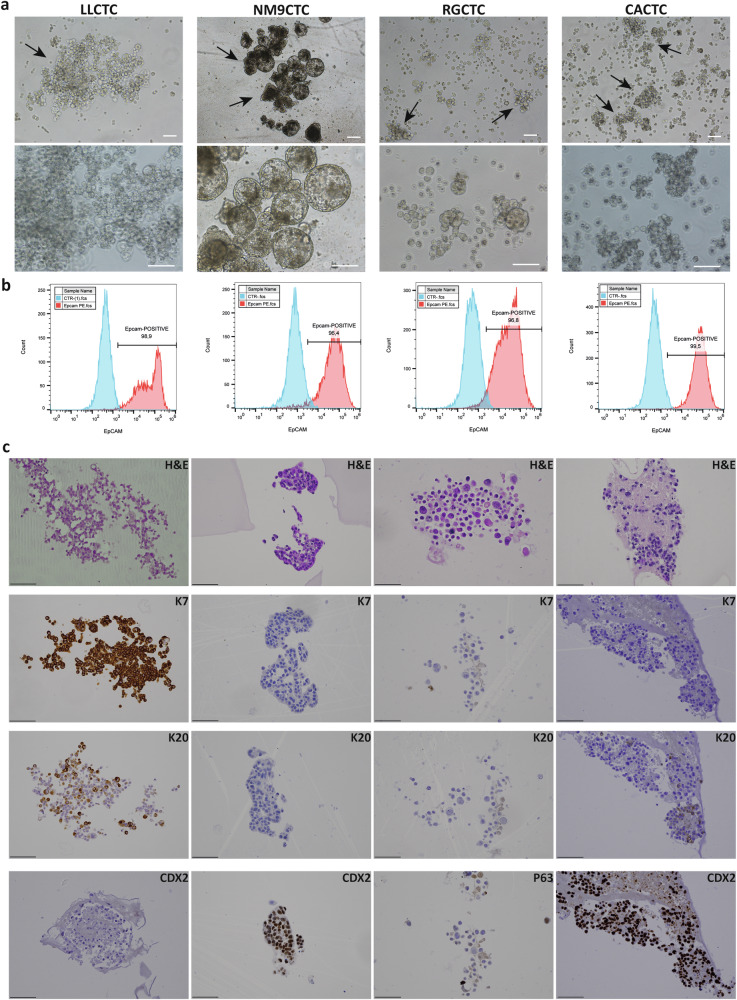
Morphological and phenotypic characteristics of cultured CTCs. **a** Representative images of the four CTC-derived cell lines. Tumoroid structures can be noticed in all cell lines (black arrows). 5x (upper) and 10x (lower) magnification, phase contrast, scalebar 100 µm. **b** FACS analysis of EpCAM-positive cell populations (red curve) and negative control (cyan curve) The percentage of EpCAM+ cells is indicated. **c** Histological characterization of CTC-derived cell lines. Panels show cellular staining with hematoxylin/eosin (H&E), cytokeratin-7 (K7), cytokeratin-20 (K20), caudal type homeobox 2 (CDX2) and tumor protein p63.

Interestingly, the four cell lines display distinctive growth behavior in-vitro. LLCTC cells initially form cell chains which fold in a sheet-like monolayer and then stratify in a more complex structure. NM9CTC cells create sealed layers and form small cyst-resembling structures, very similar to an organoid of esophagus, with the apical side of the polarized epithelium facing the lumen filled of tumor cells. They both form weakly aggregated structures, and many single cells grow in suspension (Fig. 1a).

The epithelial status of the proliferating cells was verified testing EpCAM as epithelial-specific marker using FACS analysis. All four cell lines were strongly EpCAM-positive: LLCTC 98.9%, NM9CTC 96.4%, CACTC 99.5%, RGCTC 96.8% (Fig. 1b).

CTC cell lines recapitulate the histological features of the original tumors (Fig. 1c); as confirmed in-blind by the pathologist. The two cell lines derived from adenocarcinomas (LLCTC and NM9CTC) show epithelial-like and mucinous-like structure. Signet ring-like cells are present in LLCTC structures, all cells have strong positivity for CK7, CK20 signal is patchy, and CDX2 is absent. NM9CTC cells, on the other hand, show features distinct from the other CTC cell lines: a cuboidal layer lacking mucinous or signet-ring cells; all cells are negative for both CK7 and CK20, and highly positive for nuclear CDX2. RGCTC and CACTC cell line morphology was confirmed to be esophageal squamous cell carcinoma and diffuse gastric carcinoma, respectively. RGCTC cells are negative for CK20 and p63, while some cells are strongly positive for CK7. In contrast, CACTC cell line appears negative for CK7, patchy for CK20 and strongly positive for CDX2.

### CTC-derived cell lines behavior in a zebrafish model

Zebrafish emerged as a useful vertebrate model system to study the metastatic cascade in vivo [33–35] we tested the invasiveness of CTC-derived cell lines in this in-vivo model. In a endothelium-EGFP-labelled zebrafish embryo (Tg(Fli1a:EGFP)) we injected fluorescently labelled CTC-derived cells in perivitelline space. Tumor cell invasion was followed 24- and 48 h post-injection by confocal microscopy.

**Fig. 2:**
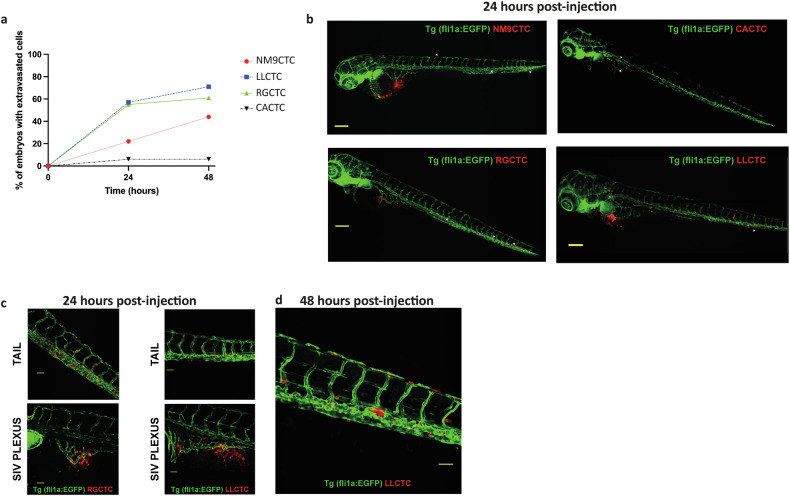
Invasive competence of CTC-derived cell lines in in-vivo zebrafish model. **a** Temporal monitoring of the percentage of embryos with CTC intravasation. **b** Representative images of engrafted zebrafish with tumor cells shed in the circulation 24 h post-injection (**c**) Representative images of tumor cell contacts with the circulatory system near the site of injection. At 24 h, sprouting vessel formations are visible, in addition to circulating CTCs. **d** Representative image of metastases formation 48 h post-injection. Green: zebrafish endothelial cells, red: CTC-derived tumor cells.

Three out of four cell lines (LLCTC, RGCTC and NM9CTC) showed invasive ability in zebrafish. The invasive competence of the cells was calculated as the fraction of fishes with tumor cells in the circulatory system. After 24 h from injection, we detected 12/21 positive zebrafish embryos in LLCTC, 10/18 in RGCTC, and 2/9 in NM9. At 48 h post-injection the number of positive zebrafish embryos increased to 15/21 ( + 3) in LLCTC, 11/18 ( + 1) in RGCTC and 4/9 in NM9 ( + 2) (Fig. 2a, b).

Timelapse experiments (24–39 h post-injection) showed that tumor cells moved within the bloodstream both passively and actively via interactions/adhesion with the endothelial layer (movie 1).

We noticed sprouting vessel formation occurred in the subintestinal vessels (SIV) area 24 h after injection (Fig. 2c). The characteristic feature of these vessels was their directly contact with the tumor cells, suggesting the intravasation of CTC-derived cell lines through vessel formation. At 48 h post-injection micrometastases localized mainly in the tail were also visible (Fig. 2d).

### Genomic and transcriptomic analyses of CTC-derived cell lines

**Fig. 3:**
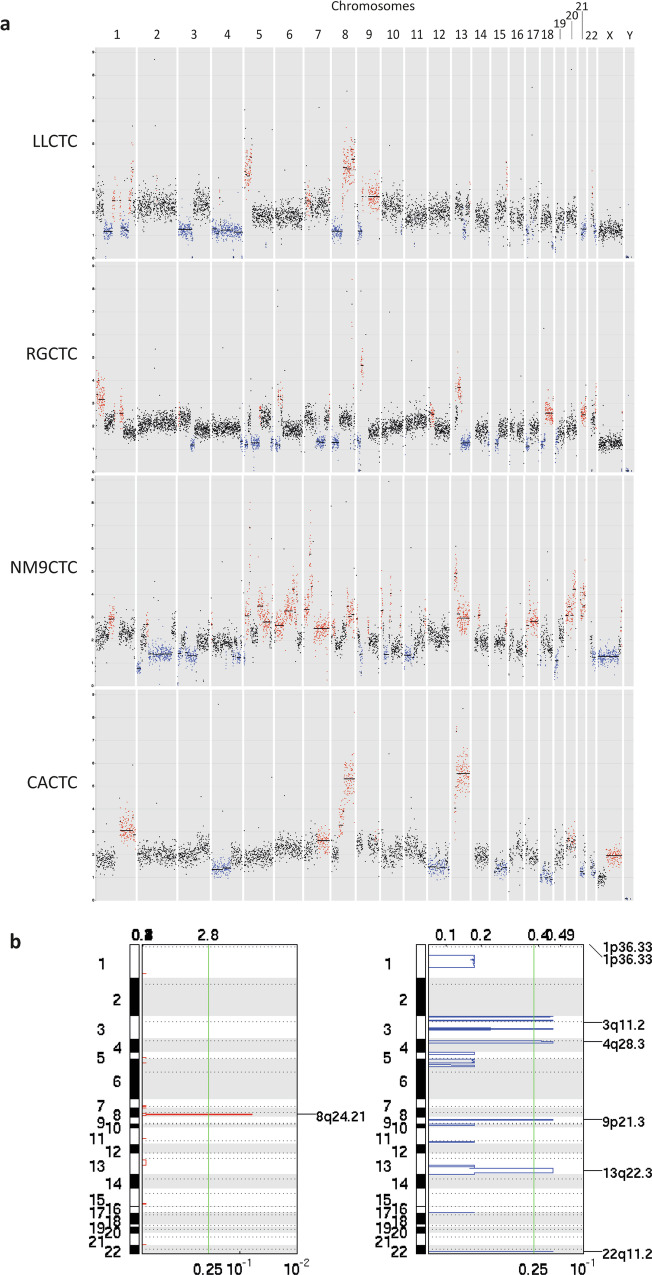
Genomic landscape of the four CTC-derived cell lines. **a** Graphical representation of CTC cell line copy number aberration (CNA) distribution across all chromosomes. Red dots represent amplifications, blue dots represent deletions (**b**) Significant copy number gains (red) and losses (blue) detected by GISTIC 2.0 (shown as peaks) shared between the cell lines.

To characterize the CTC-derived cancer cell lines, we performed a genomic analysis on the four CTC-derived cell lines revealing a wide spectrum of copy number aberrations (CNAs) (Fig. 3a). GISTIC analysis highlighted recurrent chromosomal aberration shared among most cell lines (Fig. 3b). In particular gain of chromosome 8q24.21 containing *MYC* gene [36] and loss of chromosomes 1p36.33, 3q11.2, 4q28.3, 13q22.3, 22q11.22, 1p36.33 and 9p21.3 which contain, among others, the tumor-suppressor genes CDKN2A/B and a type I IFN gene cluster [37], was also shared among most cell lines.

**Fig. 4:**
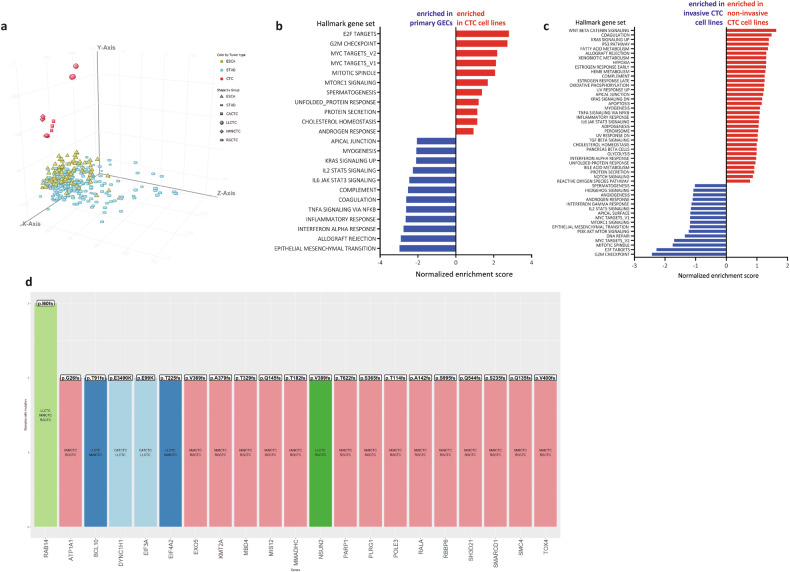
Gene expression analysis of the four CTC-derived cell lines. **a** Unsupervised principal component analysis (PCA) of the gene expression profiles of 4 CTC cell lines (each one in triplicate) and TGCA esophageal (ESCA) and stomach (STAD) cancer samples. **b** Hallmark GSEA comparison of CTC-derived cell lines versus TGCA dataset of GEC primary tumors showing normalized enrichment scores for the represented genesets (FDR < 25%, *p*-value < 0.01). Red: genesets enriched in CTC cell lines; blue: genesets enriched in primary GECs **c** Hallmark GSEA comparison of in-vivo non-invasive CTC-derived cell line (CACTC) versus in-vivo invasive CTC-derived cell lines (RGCTC, LLCTC and NM9CTC) showing normalized enrichment scores for the represented genesets (FDR < 25%, *p*-value < 0.01). Red: genesets enriched in non-invasive CTC cell line; blue genesets enriched in invasive CTC cell lines (**d**) Histogram representing somatic mutations detected in at least two cell lines. The different colors indicate different groups of cell lines.

To investigate whether the CTC cell lines had a transcriptional profile distinct from primary tumors, we obtained their transcriptomic profile and compared the CTC-derived cell lines transcriptome with that of non-pretreated resected esophageal (ESCA) and stomach (STAD) cancer samples available in TCGA database. Principal component analysis (PCA) revealed that CTC samples have a distinct gene expression profile and in particular, three CTC-derived cell lines clustered together (RGCTC, CACTC and NM9CTC) and LLCTC cell line diverged the most (Fig. 4a).

This observation led us to investigate whether there were distinct transcriptional programs in CTC and primary tumors from the same origin (Fig. 4b). Expression profiles obtained from ESCA/STAD-TCGA dataset and from CTC-derived cell lines were analyzed with GSEA using “hallmark gene sets” (h.all.v2023.1.Hs.symbols.gmt). GSEA analysis highlighted 11/50 gene sets enriched in CTC-derived cell lines (10 gene sets are significant at False Discovery Rate, FDR < 25%) and 39/50 gene sets enriched in ESCA/STAD dataset (35 gene sets are significantly enriched at FDR < 25%). Especially compelling are the gene sets enriched in CTC cell lines, which include HALLMARK_E2F_TARGETS, HALLMARK_G2M_CHECKPOINT, HALLMARK_MYC_TARGETS_V2 all with FDR 0.0 and *p*-value < 0.01. Equally meaningful are the top-three gene sets enriched in ESCA/STAD, including HALLMARK_INTERFERON_GAMMA_RESPONSE, HALLMARK_EPITHELIAL_MESENCHYMAL_TRANSITION and HALLMARK_ALLOGRAFT_REJECTION all with a FDR 0.0 and *p*-value < 0.01.

Five of the top 50 in genes differentially expressed CTCs compared to ESCA/STAD samples (Fig. S1) resulted highly expressed in all the four CTC cell lines: PCF11-AS1 (RNA gene, affiliated with the lncRNA class), MIR7161, SFT2D3 (involved in fusion of retrograde transport vesicles derived from an endocytic compartment with the Golgi complex), DND1 (encoding a protein that binds microRNA-targeting sequences of mRNA, inhibiting microRNA mediated repression) and H3C2 (a histonic protein).

The distinct in-vivo invasive properties of the CTC-derived cell lines prompted us to investigate their specific transcriptional programs. GSEA gene sets positively enriched in non-invasive CACTC cell line resulted: HALLMARK_WNT_BETA_CATENIN_SIGNALING, HALLMARK_COAGULATION, HALLMARK_KRAS_SIGNALING_UP. While GSEA gene sets enriched in invasive CTC cell lines were 11 and the top three were: HALLMARK_G2M_CHECKPOINT, HALLMARK_E2F_TARGETS, HALLMARK_MITOTIC_SPINDLE (Fig. 4c).

Using RNA sequencing data, we identified somatic mutations in all CTC cell lines. The number of non-synonymous or frameshift mutations ranged from 26 in CACTC to 46 in LLCTC, 70 in RGCTC and 106 in NM9CTC cell line; all the exonic variants are listed in Supplemented Table S1.

We found mutations in known esophageal and gastric cancer genes including *TP53*, *ARID1A* and *PIK3CA* [4]. We also detected mutations in genes involved in DNA repair (*PARP1*, *BCL-10*, *CCNB*, *MBD4*, *HMGB1*), histone deacetylases (*HDAC9*, *HDAC7*) and *HIF1A*.

Specific mutations shared between two or three cell lines are reported in Fig. 4d. We found no common mutations in all four lines, nor distinctive for tumor type, but interestingly the p.I60fs *RAB14* mutation is present in the three invasive CTC cell lines (LLCTC, RGCTC and NM9CTC).

### Characterization of extracellular vesicles secreted by CTC-derived cell lines

We hypothesized that CTC-secreted extracellular vesicles (EVs) could be relevant to the process of metastasis.

We investigated this hypothesis by comparing the characteristics of EVs secreted by the four CTC cell lines with the EVs released by two commercial tumor cell lines. Specifically, OE-33 and NCI-N87 derived from esophageal [38, 39] and gastric cancer [40], respectively.

**Fig. 5:**
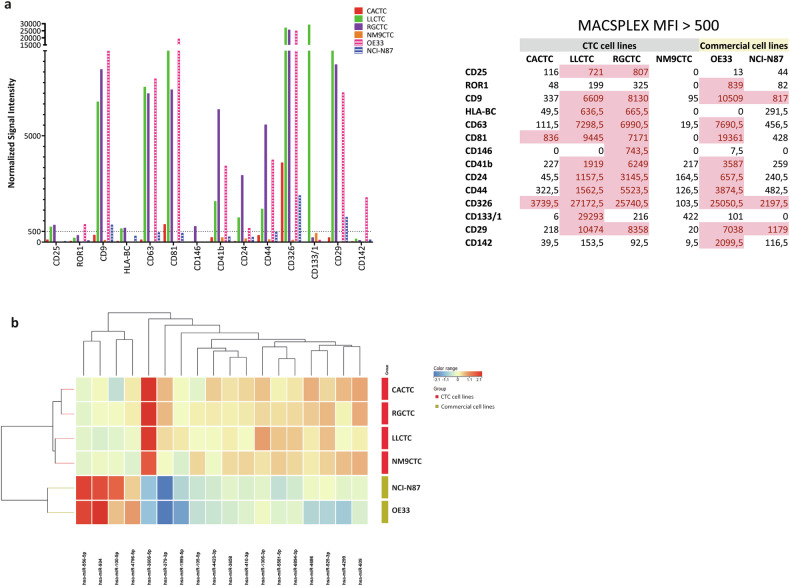
Characterization of extracellular vesicles secreted by cell lines. **a** Phenotypic characterization of extracellular vesicles isolated from supernatant of exhausted culture media of cell lines obtained by FACS analysis. Histograms represent the mean fluorescence intensity (MFI) of the detected membrane proteins with values > 500 in at least one of the samples. MFI values are reported in the side table. **b** Cluster analysis and heatmap representation of CTC and commercial GEC cell lines based on the 14 miRNAs contained into EV and differentially expressed between the two groups.

To maximize the EV production, cells were cultured in the 3D scaffold Alvetex. EVs were enriched through 70 nm qEV10 Size Exclusion Columns. The estimated number/mL of EVs was determines by nanoparticle tracking analysis (NTA). We did not observe any difference in the number of EVs between the six samples, all ranging from 10^9^ to 10^10^ particles/mL (Fig. S2). We evaluated the EV phenotypic traits by using FACS analysis and MACSPlex exosomes kit. We observed that all the EV samples were strongly CD9/CD81/CD63 positive and retained the CD326 marker of epithelial origin. Although we did not detect a phenotypic marker present only on CTC-derived EVs, we noticed that HLA-BC, CD41b, CD24, CD44 and CD29 were detected at higher levels in LLCTC and RGCTC CTC-derived cell lines (Fig. 5a).

We investigated the EV cargo by comparing the miRNA profile of CTC-derived and tumor-derived EVs. We found 18 miRNAs differentially expressed in the two EV types; interestingly, 14 miRNAs are carried specifically in EVs secreted by CTC cell lines (Fig. 5b), on the contrary miR-555-5p, miR-934, miR-100-5p and miR-4796-5p were detected only in tumor cell lines (Fig. 5b). The target enrichment analysis of these miRNAs conducted with Metacore showed an enrichment in miRNA validated target genes belonging to YAP1, BRG1/AKT1 and TCF8/HDAC2 networks (Fig. S3).

### Characterization of cellular components in co-cultures (bioreactors)

**Fig. 6:**
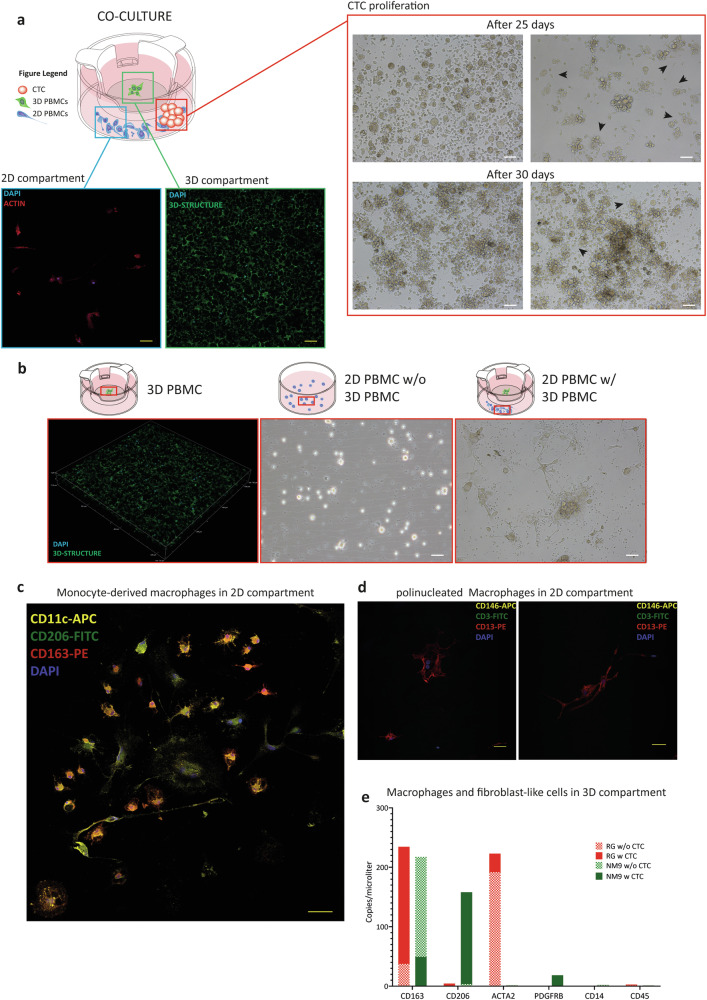
Characterization of immune cell differentiation in co-cultures. **a** Schematic representation of the co-culture (bioreactor): Isolated PBMCs were seeded in the 3D compartment, while enriched CTCs are seeded in-suspension (2D) with residual PBMCs. After 30 days, proliferating CTCs are visible (brightfield images) and differentiated monocytes are evident both in the 3D scaffold (cells are immune-stained with DAPI, blue) and at the bottom of the ultra-low attachment well (cells are stained with phalloidin, red, and counterstained with DAPI, blue). **b** Leukocyte differentiation at the bottom of the well is dependent on the presence of differentiated leukocytes in the 3D scaffold. When seeded without the 3D compartment, leukocytes do not differentiate (adhere at the bottom of the ultra-low attachment well) (2D PBMC w/o 3D PBMC), but rather persist in-suspension and then die in about 30 days. Leukocytes in 2D compartment adhere and differentiate only in the presence of differentiated leukocytes in the 3D compartment (2D PBMC w/ 3D PBMC). **c** Representative immunofluorescence staining of differentiated leukocytes in the 2D-compartment indicating differentiated monocytes with a mixed population of macrophages M2-like profile (CD206 + /CD163 + ) and M1-like profile (CD11c + ), nuclei are in blue. **d** Representative images confirming the differentiation of monocytes into macrophages in patient co-cultures, Cells multinucleation were verified through the cell-cell fusion marker CD13 (red), CD146 (yellow) as a monocyte-derived subtype of macrophages and CD3 (green) to discriminate any residual T-lymphocyte. **e** Expression of cell type or lineage-specific genes including ACTA2, PDGFRb (fibroblasts/myofibroblasts), CD163, CD206 (macrophages) and CD14, CD45 as monocyte lineage, using Digital PCR. Expression analysis revealed a mixed population of CD163 + /CD206+ macrophages and fibroblast-like cells ACTA2/PDGFRb+ on 3D compartments seeded with patient’s PBMCs (with, plain colours, or without proliferating CTCs checkered pattern).

Since four CTC cell lines were successfully established only in the co-culture setting, while 2 out of 6 failed to grow, we decided to investigate the features of the PBMC populations in “successful” vs. “failing” co-cultures. We recognized differentiated leukocytes both in the 2D and 3D compartment of the bioreactor (Fig. 6a).

At first, we investigated whether the leukocyte differentiation was compartment dependent. Using the same in-vitro conditions, we seeded healthy donor PBMCs in separated compartments. Specifically, we seeded a controlled number of leukocytes in the 2D compartment in the absence of cells in the 3D and vice versa, and a complete co-culture condition where leukocytes were present in both compartments (Fig. 6b).

After 20 days of culturing, no adhering leukocytes were detectable in the single 2D culture condition. We identified adherent leukocytes in the single 3D culture and in the complete co-culture conditions, using confocal microscopy by nuclei staining. Only in the complete co-culture condition, adherent leukocytes in the 2D compartment were present. These cells did not proliferate but rather showed a tight adhesiveness on an immobilization-inhibiting surface (ultra-low attachment) suggesting a differentiation program activation leading to matrix production.

Adherent leukocytes in the 2D compartment of the co-culture showed a mixed morphology: there were large cells with an egg-like shape with numerous folds and projections, elongated bipolar cells, cells exhibiting lamellopoidia, smaller rounded cells with cytoplasmic processes with a star-shaped morphology (Fig. 6a).

The morphological appearance and ability to adhere on an ultra-low plastic led us to hypothesize that they derived from a monocyte population differentiated into macrophages. Indeed, confocal microscopy analysis showed that the cells in the 2D compartment are myeloid-derived (CD14 + ) with a macrophages M2-like profile (CD206 + /CD163 + ) positive for HLA-DR and macrophages M1-like profile (CD11c + ), while negative for dendritic markers CD1a, CD80, CD83. The CD11c/CD206/CD163 signature was detected also in the patient-derived leukocytes cultured the 2D compartment (Fig. 6c, Fig. S4).

As cells showed multinucleation, we verified their positivity for CD13, a cell-cell fusion marker, CD146 as a monocyte-derived subtype of macrophages and CD3 to discriminate any residual T-lymphocyte. Patients’ leucocytes in the 2D compartment were actual multinucleated cells positive for cell-cell fusion marker CD13, negative for CD146, and negative for CD3 (Fig. 6d).

Due to the high autofluorescence of the scaffold, fluorescence microscopy could not be used for the identification of leucocyte differentiation in the 3D compartment, therefore we extracted the RNA from these cells and employed a Digital PCR testing to assess the expression of cell type or lineage-specific genes including that of fibroblasts/myofibroblasts (ACTA2, PDGFRb), macrophages (CD163, CD206) and monocyte (CD14, CD45) lineages (Fig. 6e). We tested the expression of these genes in the co-culture in the presence or absence of CTCs. The results showed that 3D leukocytes completely loose the lineage markers (CD45 and CD14) and differentiate into a mixed population of macrophages (CD163 + /CD206 + ) and fibroblasts (ACTA2 + /PDGFRb + ).

### Secretome composition of co-cultures

To gain insight into cell-to-cell crosstalk within the tumor microenvironment, we investigated the secretome (cytokines and EVs) of the co-cultures.

We investigated the cytokine composition secreted by the leukocyte populations grown in separate compartments and in the co-culture system by performing an 80-cytokine array on supernatants. This analysis showed that the two distinct compartments secrete specific cytokines, and that there is a specific cytokine-based crosstalk within the complete co-culture (Fig. S5). Since leukocytes in the 3D compartment drive the differentiation of those in the 2D compartment, we identified ENA-78 as a cytokine secreted exclusively in the isolated 3D compartment and in the complete co-culture. Nine cytokines were detected only in the complete co-culture: GRO, GRO alpha, IL-6, I-309, MCP-1, MCP-2, MCP-3, MCP4 (Fig. S5a). Then, we analyzed the same cytokine panel in the bioreactor with CTCs; we did not identify any additional cytokine discriminating between the presence and absence of proliferating CTCs (Fig. S5b).

To evaluate the phenotype of EVs produced in the co-cultures, we performed the multiplex assay MACSPlex which allows the detection of 37 EV membrane proteins including EV markers CD9, CD63 and CD81.

We first compared the MACSPLEX profile of GEC patient-derived PBMC co-cultures with that of a healthy donor PBMC co-culture considering a 100 median fluorescence intensity (MFI) as a robust positive signal [41]. All the samples were positive for exosome markers CD9, CD63 and CD81. Phenotypic profiles are very different between patients and healthy donors, as EV markers of the latter are typical of cells with immunomodulatory activity, specifically: HLA-DR, CD40, CD11c, CD29 (above the threshold of 500 MFI). CD41b was found to be unique EV marker of PBMC co-cultures of gastroesophageal cancer patients, and no signal was detected in healthy donor EVs (Fig. S6a).

Comparing the phenotypic profile of EVs secreted in co-cultures of EGC PBMCs in the presence or absence of proliferating CTCs (Fig. S6b), we confirmed the positivity for the exosomal markers CD9, CD63, CD81 and the presence of epithelial marker EpCAM on EVs in co-cultures with proliferating CTCs. In contrast, CD11c (dendritic cells) and MSCP1 were detectable at high ( > 100 MFI) levels in EVs in co-cultures without proliferating CTCs.

Since microRNAs are one of the main components of EVs, we compared the miRNA content of EVs in successful CTC co-cultures and failing CTC co-cultures. Specifically, we compared the miRNA profiles of EVs secreted in: healthy donor co-culture (*N* = 3), CTC-failure co-culture (*N* = 2), CTC-success co-culture, with growing CTCs (*N* = 2) and without CTCs (*N* = 2), and CTC-derived cell lines expanded after the co-culture (*N* = 4). Comparing the miRNA profile of all EV samples (PCA analysis (Fig. S7)) we observed that the EVs of successful co-cultures have a microRNA content that differs from failing/healthy co-cultures or co-cultures without CTCs and become more similar to EVs released by tumor cell lines. This content is maintained only when the CTC cell line grows with the support of the co-culture system.

We identified five miRNAs delivered in EVs of CTC-success co-culture with CTCs, as shown in the supervised heatmap where this class of samples clustered independently (Fig. S8). METACORE analysis showed that these miRNAs target genes that belong to NANOG, p53 and STAT1 pathways (Fig. S9).

## Discussion

In this study, we exploited the interaction between cells of the immune system and CTCs to sustain CTC proliferation. Extending Virchow’s hypothesis by Balkwill and Mantovani stating that certain types of inflammation are the “fuel that feeds the flames” [42], we established in-vitro co-cultures between the host immune cells and CTCs from the same patient. In this context, the co-culture generates a microenvironment which sustain CTC indefinite proliferation. Using an optimized co-culture system (bioreactor) we obtained 4 CTC cell lines starting from EGC CTC enriched from the peripheral blood; which were not able to grow when cultured using the same conditions, but without the immune microenvironment.

The 4 CTC-derived cell lines replicate the structure and morphology of the tumor of origin, as well as EpCAM positivity, the marker of choice for CTCs of epithelial origin. Despite the technical difficulties associated with injecting compact tumoroids in zebrafish embryos, in-vivo zebrafish model intravasation and extravasation experiments showed that three out of 4 cell lines have invasive characteristics. Indeed, they enter the bloodstream within 24 h (through sprouting vessel formation) and originate micro-metastases within 48 h after injection. This unexpected result further consolidates the consensus regarding the heterogeneity of CTCs [10, 43, 44] not only in phenotypic aspects [16], but also in behavioral patterns within the metastatic cascade.

By investigating the genetic features of the CTC-derived cell lines, we verified that they bear amplification of chromosome 8q24.21 which is known to contain the oncogenic gene *MYC* [2, 4]; of particular interest are also the chromosomal deletion of 9p21.3 containing the tumor suppressor gene *CDKN2A/B* and the IFN type I gene cluster. These amplification/deletion patterns were previously described in colon stem cells generating CTCs in mouse models [36]. In addition, in a recent study, Iriondo et al. described that suppression of type I IFN signaling by hypoxic memory increases the metastatic capacity of tumor cells, including cell lines derived from breast cancer CTCs [45]. These recurring genetic findings strongly suggest that these traits represent a CTC selective survival advantage in the bloodstream through amplification of oncogenic genes and deletion of mediators of immune evasion genes. These genetic characteristics are well reflected in the transcriptomic profiles of the CTC-derived lines, as they diverge from transcriptomic profiles of the gastroesophageal cohort of the Cancer Genome Atlas (TCGA). In particular, gene sets related to E2F, G2M and MYC targets emerge as upregulated, while gene sets related to antibody recognition such as gamma-interferon responses, EMT, allograft rejection emerge as downregulated, suggesting that CTCs are immune-evading tumor cells.

By exploring the differences in the transcriptional profile of non-invasive and invasive CTC cell lines, we observed an enrichment in gene sets of Wnt/beta-catenin, coagulation and K-RAS signaling in non-invasive cell lines. The four cell lines have a significant number of mutations typical of gastroesophageal cancer (*TP53, ARID1A, PI3KCA*) [46], but also in genes involved in DNA repair [2] and histone deacetylase activity in cancer [47]. Despite the high mutational rate, we did not identify common mutations; although, the frameshift mutation pI60fs in RAB14 was shared among all three invasive CTC cell lines. This mutation has not been described before, but recent findings reported *RAB14* as an oncogene that is involved in the trafficking of tumor derived EVs by enhancing the angiogenic potential of endothelial cells [48].

This result led us to investigate the EVs secreted by CTCs. CTC-derived EVs retain a strong expression of EpCAM epithelial marker and while no phenotypic difference was detectable from EVs from commercial EGC cell lines, we observed a change in the pattern miRNAs delivered as CTC cell line cargo. In particular, 14 miRNAs are distinctive of CTC derived EVs and the pathway enrichment analysis showed that these miRNAs are involved in the regulation of YAP, BRG1-AKT1, TCF8-HADAC2 networks. MiR-3606-5p, the miRNA most enriched in CTC-EVs is known to regulate TGF-beta receptor type II [49]. Interestingly, miR-379-3p and miR-199b-5p are reported to act as proliferation inhibitors in tumors and cell lines [50] while miR-199-5p cargo of CAF-derived EVs promoted gastric tumorigenesis in vivo through the pathway of AKT1 and mTORC1 [51]. The miR-410-3p and miR-4299 were implicated in promoting EMT/radioresistance via PTEN/PI3K/mTOR axis [52] proliferation and metastasis [53].

Since CTCs grew only in the bioreactor, in co-culture with immune cells, we wanted to investigate the composition in terms of cell type and secretome of the bioreactor. Within the co-cultures, we observed the formation of a microenvironment mainly composed by two monocyte-derived cell types: macrophages and fibroblasts. Our co-cultures revealed that their differentiation occurs in a hierarchical order: differentiated monocytes on the 3D compartment prompts monocytes differentiation on the 2D compartment.

In particular, the 3D compartment is composed of a mixed population of CD206 + /CD163+ macrophages and fibroblasts expressing ACTA2/PDGFBRbeta which are known as distinctive for cancer associated fibroblasts [54, 55]. The presence of this mixed population in the 3D compartment, induces the differentiation of monocytes in the underlying co-culture (2D compartment) into M2 (CD206 + /CD163 + ) and M1 (HLA-DR + /CD11c + ) macrophages; furthermore, some of these cells were polynucleated probably as a result of cell-cell fusion processes, as suggested by CD13 positivity [56].

The microenvironment generated by these cell populations is what induced CTC proliferation, as supported by the inability of CTCs to grow outside the bioreactor.

This effect is in line with the dormancy-awakening mechanism described in previous works [57]. Since the dormancy phenomenon of “temporary mitotic and growth arrest“ [58] is reversible [59], dormant disseminated CTCs [60, 61] may awaken as a consequence of their interaction with factors produced by activated endothelial cells [62], stromal cells [63], osteoclasts [64] neutrophils through neutrophil-derived extracellular fibers (NET) [65], and the most studied myeloid cells, like monocyte-derived macrophages [66, 67].

The CTC proliferation latency (20-30 days) suggests its dependency from monocytes differentiation and the presence of soluble factors produced by these cells.

The cytokines secreted in the bioreactor (MCP-1/CCL2 [68, 69], ENA-78/CXCL5 [68, 70]) have been implicated in the cross-talk between cancer associated fibroblasts (CAFs) and tumor associated macrophages (TAMs) 71, 72 Within the co-cultures, we find additional cytokines that contribute to the establishment of an inflammatory tumor microenvironment, such as SPP1 [73], MCP-3 [68], RANTES [68, 74], IL-8 [69, 75].

Of note, we did not identify distinctive cytokines released only in the presence of proliferating CTCs, thus suggesting that these microenvironment components are driven specifically by the immune cell compartment.

Interestingly, we found differences in the phenotypic profile of EVs derived from patients’ co-cultures: EVs in GEC patient-derived bioreactors express CD41b while EVs in bioreactors with PBMC from healthy donors display an immuno-modulatory phenotype (HLA-DR/CD40/CD11c/CD29).

We also identified 5 unique miRNAs in EVs of bioreactors with proliferating CTCs, but not present in EVs from failing/healthy co-cultures or bioreactors without CTCs, thus suggesting that the crosstalk between fibroblasts, macrophages and CTCs occurs mostly through the EV exchange. In addition, this EV content is maintained only when the CTC cell line grows with the support of the co-culture system, thus suggesting the relevance of the tumor-immune cell crosstalk in the very early phases of the metastatic process.

Metacore analyses showed that these miRNAs are involved in the regulation of NANOG, p53 and STAT1 pathways. Among these miRNAs, mir-483 is located in the intronic sequence of the IGF2 gene, has been observed to induce in-vitro the proliferation of hepatocellular carcinoma cells [76, 77]

Our data indicate that an in-vitro inflammatory environment [42] generated in co-cultures with autologous immune system cells is able to induce CTC proliferation, leading to the successful establishment of CTC-derived cell lines. The inflammatory environment is determined by the progressive monocyte differentiation toward CAF-like fibroblasts and M2/M1 macrophages, as the result of the secretion of specific cytokines. These cells communicate with CTCs through the release of EVs, whose miRNA cargo changes as a consequence of the crosstalk. Specifically, 5 miRNAs contained in EVs are most likely involved in the initial in vitro proliferation and survival of CTCs. The CTC-derived cell lines are able to independently form 3D structures that resemble the tumor of origin, retain positivity to EpCAM, and genetically exhibit 8q24.21 amplification containing the *MYC* oncogene. From a transcriptional point of view, these CTC cells lines show immune-evasion characteristics that likely drive their survival advantage in the bloodstream.

## Methods

### CTC and PBMC isolation and cell line establishment

Patients with plurimetastatic gastrointestinal cancer were enrolled at the IRCCS Istituto Romagnolo per lo Studio dei Tumori (IRST) “Dino Amadori” (Italy) between 2015 and 2019 (*N* = 24). From each patient we collected about 20 mL of peripheral blood. The study was conducted under Romagna Ethics Committee (CEROM) of Meldola approval (ref. B063) according to the recommendations of the declaration of Helsinki. All patients provided informed consent prior to blood acquisition.

About 20 mL of peripheral blood samples was drawn into a EDTA vacutainers (BD Diagnostics, Franklin Lakes, NJ, USA) and processed within 3 h.

The workflow for cells co-culturing is outlined in Fig. S10.

#### PBMC preparation for culture

PBMCs were isolated from 3 ml of peripheral bloodusing density gradient centrifugation via Ficoll-Paque(GE Healthcare, Pittsburgh, PA, USA). Peripheral blood was diluted (1:2) with sterile DPBS 1X (GIBCO) and layered gently over Ficoll-Paque. After centrifugation (1200 *g*, 30 min without break) PBMC buffy coat was enriched at the interphase between plasma and Ficoll-Paque. Buffy coat was carefully aspirated without disturbing red blood cells layer, isolated PBMCs were washed with DPBS X twice and counted for seeding.

#### CTC preparation for culture from 18-20 ml of peripheral blood

CTCs were enriched using negative depletion reagent with a minimal handling protocol in order to minimize CTC loss. Peripheral blood was firstly incubated with RosetteSep™ CTC Enrichment Cocktail (StemCell Technologies, Vancouver, Canada) (using 40 ul of RosetteSep™ solution per ml of blood instead of 50 ul/ml) for 20 min at room temperature. During this step the tetrameric antibody complexes targeting CD2, CD16, CD19, CD36, CD38, CD45, CD66b and glycophorin A on erythrocytes are formed.

Following incubation, blood was diluted (1:2) with sterile DPBS1X and layered gently on the top of the insert barrier of a SepMate™ tube (StemCell Technologies, Vancouver, Canada) previously loaded with 15 ml of Ficoll-Paque (above the insert) and centrifuged at 1200 *g* for 10 min brake on. During centrifugation tetrameric antibody complexes of unwanted cells together with erythrocytes (serving as anchor) are pelleted at the bottom of the tube. SepMate top layer, containing enriched CTCs, was poured off into a new 50 ml tube. Enriched CTCs were washed with DPBS1X and resuspended with serum-free culture medium for co-culture.

Serum-free culture medium comprised DMEM/F12 Glutamax (GIBCO, Life Technologies, Paisley, UK) supplemented with 15 mM HEPES (Life Technologies), 25% IntestiCult™ Organoid Growth Medium (Stem Cell Technologies), Heparin Solution (Stem Cell Technologies), 1% penicillin-streptomycin (GIBCO), 20 ng/mL human recombinant Epidermal Growth Factor (EGF; Peprotech, London, United Kingdom) and 20 ng/mL human recombinant Fibroblast Growth Factor (FGF; Peprotech).

#### Assembly of the co-culture

The co-culture system consists of 3 compartments: a well-insert with Alvetex® scaffold technology (Reprocell; MD, USA) - a 200 ul thick polystyrene scaffold, the bottom area of the well composed by an ultra-low attachment plastic surface (2D) and in the middle the in-suspension area.

The assembly of the co-culture involved the in-suspension seeding of enriched CTCs, following a 1 h PBMCs seeding on 3D well-insert in an ultra-low attachment 6-well plate.

Prior to cell seeding, Alvetex® scaffold well insert was rendered hydrophilic by washing it with a sterile solution of 70% ethanol, followed by a wash step with DPBS1X.

To prevent the 3D scaffold from drying out, the co-culture system was filled with culture medium to make the 3D scaffold wet. 106 PBMCs (50ul volume) were seeded carefully directly onto the center of the Alvetex® scaffold. Enriched CTCs were in-suspension seeded below the well insert. Co-cultures were maintained in a humified incubator at 37 °C with 4% O2 and 5%CO2.

### Immunohistochemical and immunophenotypical analysis

CTC-derived cell lines structures were encapsulated in a 1% Agar (Life Technologies) domes, to preserve their 3D-organization. All the domes were fixed in 10% formalin and paraffin embedded, with 4 µm sections cut for staining. Antigen retrieval and antibodies staining were performed on the fully automated slide stainer Ventana (Ventana Medical Systems Inc, Roche AG) according to manufacturer’s instructions using antibodies directed toward: Cytokeratin-7 (CONFIRM anti-Cytokeratin 7 (SP52) Rabbit Monoclonal Primary Antibody, Roche Ventana), Cytokeratin-20 (CONFIRM anti-Cytokeratin 20 (SP33) Rabbit Monoclonal Primary Antibody, Roche Ventana), CDX-2 (CDX-2 (EPR2764Y) Rabbit Monoclonal Antibody, Roche Ventana), p63 (VENTANA anti-p63 (4A4) Mouse Monoclonal Primary Antibody, Roche Ventana) and counterstained with Hematoxylin II.

Flow cytometric analysis was performed using a FACS-Canto flow cytometer (Becton Dickinson, Franklin Lakes, NJ, USA). A total of 10^6 events per sample were acquired using FACSDiva software (Becton Dickinson), events were acquired at the medium rate (approximately 200 events/seconds) background fluorescence was estimated by substituting primary antibody with specific isotype control. The following primary antibodies were employed for 15 min in the dark at room temperature according to manufacturer’s instructions: CD326-FITC (EpCAM) Antibody, anti-human, REAfinity™ (Mitenyi Biotec GmbH, Germany) and FITC conjugated REA Control Antibody, human IgG1, REAfinity™ (Mitenyi Biotec).

Fluorescence Microscopy were performed on fixed cells with 4% paraformaldehyde, samples were blocked with 2% Bovine Serum Albumin (BSA, St. Louis, MO, USA), stained with 1 µg/mL 4′,6-diamidino-2-phenylindole (DAPI, Life Technologies) and incubated overnight at 4 °C with conjugated primary antibodies. List of employed antibodies:

**Table:** 

CD206	FITC Anti-Mannose Receptor antibody [15-2]	ab270647
CD163	PE Anti-CD163 antibody [GHI/61]	ab95613
CD83	BD Pharmingen™ APC Mouse Anti-Human CD83 Clone HB15e (RUO)	551073
CD3	BD Pharmingen™ FITC Mouse Anti-Rat CD3	554832
CD13	CD13 Antibody, anti-human, REAfinity™	130-120-238
CD11c	BD Pharmingen™ APC Mouse Anti-Human CD11C Clone B-ly6 (RUO)	559877
CD14	CD14 Antibody, anti-human, REAfinity™	130-110-518
CD1a	BD™ CD1a PE Clone SK9	333167
HLA-DR	HLA-DR Antibody, anti-human, REAfinity™	130-111-789
CD80	CD80 Antibody, anti-mouse, REAfinity™	130-116-461

Cell images were captured by a Nikon Eclipse Ti2 confocal microscope (Nikon Corporation, Tokyo, Japan) using NIS Elements software (Nikon Corporation).

### CTC monitoring in Zebrafish embryo

Adult and embryo of *Tg(fli1a:EGFP)* transgenic zebrafish strain [78] were maintained and handled according to and European Directive 2010/63/EU and Italian law (D.Lgs 26/2014).

Fertilized eggs were maintained in fish water supplemented with 0.1% of Methylene Blue at 28 °C. Before handling, embryos were anesthetized with 0.02% tricaine solution (Ethyl 3-aminobenzoatemethanesulfonate salt, Sigma-Aldrich® Merck KGaA). Prior to injection, CTC cells were labeled with a red fluorescent viable dye (CellTracker™ Deep Red Dye, Invitrogen, Carlsbad, CA, USA) and resuspended with PBS at the concentration of 2.5 × 10^5^/µL. The cell suspensions were grafted into the perivitelline space close to the sub-intestinal vein (SIV) plexus of 48 hpf *Tg(fli1a:EGFP)* embryos using a pulled micropipette. 3 h post implantation, the embryos were screened to select the correctly grafted larvae. Injected embryos were maintained at 32 °C before image acquisition. Circulating cells were monitored in vivo for 24 h and 48 h by means of a confocal microscopy (Nikon Corporation, Tokyo, Japan) and images analyzed with the NIS Elements software (Nikon Corporation, Tokyo, Japan).

### Whole genome copy number analysis

For whole genome copy number aberration analysis (CNA), a cellular pellet containing approximately 1000 cells from each cell line was subjected to whole genome amplification using the Ampli1 WGA kit (Menarini Silicon Biosystems, Castel Maggiore, Italy), following the protocol provided by the manufacturer. The quality of each amplified DNA using the Ampli1 QC Kit (Menarini Silicon Biosystems). The resulting PCR products were run on a 2,5% agarose gel following and QC bands were visualized using the Chemidoc XRS System (Bio-Rad, Hercules, CA, USA). WGA products were conserved at −20 °C until further downstream analysis.

Libraries were prepared using the Ampli1 LowPass kit for Ion Torrent (Menarini Silicon Biosystems), following the protocol provided by the manufacturer. For determination of library concentration and size, Qubit™ 1X dsDNA High Sensitivity (HS) (Thermo Fisher Scientific, Waltham, MA, USA) and Bioanalyzer High Sensitivity DNA Analysis (Agilent Technologies, Santa Clara, CA, USA) were used, respectively. For sequencing, Ion chips were prepared using the Ion Chef platform and sequenced using the Ion S5 sequencing system (Thermo Fisher Sc.).

CNA calling was performed as described in [79]. Briefly, BAM files were processed by Control-FREEC [80] Mann–Whitney and Kolmogorov–Smirnov statistical tests were used to assess the statistical significance of each CNAs. We applied the Genomic Identification of Significant Targets in Cancer (GISTIC) tool [81] to identify aberrant chromosomal regions shared between the four cell lines.

### RNA extraction

RNA extraction from CTC cell lines was performed using RNeasy Plus Mini Kit (QIAGEN, Hilden, Germany). RNA extraction from differentiated PBMC was performed using TRIzol reagent (Invitrogen) according to the manufacturer’s instructions and purified using the RNeasy MinElute CleanUp kit with DNAse treatment (Qiagen). RNA concentration was evaluated using the Spectrophotometer Nanodrop-ND-1000 system (Celbio, Milan, Italy). RNA extraction from EVs was performed using the Plasma/Serum RNA Purification Mini Kit (Cat. 56100, Norgen Biotek, ON, Canada), following the manufacturer’s protocol. All samples were stored at −80 °C until further analyses.

### RNA sequencing and gene expression analysis

We conducted a short-read, stranded RNA sequencing (RNA-seq) on the four CTC-derived cell lines. RNA-seq experiments were performed using 3 biological replicates for each cell line. RNASeq libraries were generated for the four CTC-derived cell lines using Qiagen QIAseq Fast Select RNA Removal kit (Qiagen) and Qiagen QIAseq stranded total RNA library kit (Qiagen). Specifically, for library generation 500 ng of total RNA (RIN > 8) was used as a starting material. Reverse transcription, second strand synthesis, end-repair, A-addition and adapters ligation were performed as reported in the manufacturer’s instructions. Quality Control (QC) of the libraries was performed using Agilent High Sensitivity DNA kit 1000 (Agilent). RNA-Seq was performed using the Illumina NextSeq 500 Instrument (Illumina) and the NextSeq High Output kit v.2.5 paired-end flowcell (Illumina).

The RNA-seq data preprocessing and analysis workflow, specifically tailored for differential expression gene (DEG) analysis between primary tumors and circulating tumor cells, was drafted using Snakemake v.7.18.1 [82], a robust workflow management system performed in Anaconda environment v.23.1.0. Raw sequencing reads were quality checked using FastQC v.0.11.8 and filtered to remove low-quality reads and adapter sequences using Trimmomatic v.0.39. Filtered reads were aligned to the reference genome from Gencode release 41 (GRCh38.p13) using STAR v.2.7.10b with default parameters from Release 32 of the GDC mRNA quantification analysis pipeline. Gene expression levels were quantified and normalized by using DESeq2 R package [83], and differential expression analysis was performed to identify genes with significant expression changes between conditions. Gene set enrichment analysis was conducted using GSEA software [84].

### Small RNA sequencing

Libraries were prepared using the Qiaseq miRNA library kit (Qiagen) starting from 5 ul of total RNA and following the protocol for low input samples. Libraries were quantified using the Qubit dsDNA HS kit (Thermo Fisher), and quality was assessed using the Bioanalyzer DNA HS kit (Agilent). Sequencing was performed on the NextSeq550 Instrument (Illumina). Library pools were diluted to 1.5 pM and sequenced using NextSeq 500/550 High Output Kit v2.5 75 cycles flow cell (Cat. N° 20024906, Illumina, San Diego, CA, USA) on NextSeq 500 platform (Illumina). Sequencing data analysis: raw data (FASTQ) were analyzed according to the QIAseq miRNA Primary Quantification pipeline using the GeneGlobe Data Analysis Center (available at https://geneglobe.qiagen.com/us/analyze Qiagen, Hilden Germany). All miRNA UMI (Unique Molecular Identifiers) raw counts were normalized using the bioconductor R package DESeq2 (Love 2014, PMID: 25516281). Differentially expressed miRNAs were identified using GeneSpring GX software (Agilent Technologies). Top scored networks of miRNAs cargo EVs were performed using MetaCore^TM^ software (Clarivate Analytics, UK, https://clarivate.com/products/metacore/).

### Gene expression analysis with Digital PCR

Reverse transcription reactions were performed in 20 µL of nuclease free water containing 80 ng of total RNA using iScript cDNA Synthesis kit (Bio-Rad Laboratories, Hercules, CA, USA).

The resulting cDNA was stored at −20 °C. For dPCR, the chip-based QuantStudio 3D Digital PCR system (Applied Biosystems, Foster City, CA, USA) was used to determine the expression of ATCTA2, CD163, CD206, CD14, CD45, PDGFRB with B2-microglobulin as an internal control. All dPCR experiments were carried out using the chip-based QuantStudioTM 3D Digital PCR system (Applied Biosystems, Foster City, CA, USA), in accordance with the “Minimum Information for Publication of Quantitative Real-Time dPCR Experiments” (dMIQE) guidelines. All multiplex reactions mix were composed by 2X QuantStudio 3D™ Digital PCR Master Mix v2 (Applied Biosystems), 20X gene specific assay FAM and 20X gene B2 microglobulin assay VIC. In brief, chips were run in GeneAmp PCR System 9700 (Applied Biosystems) by applying the following conditions: hold at 96 °C for 10 min; 45 cycles of 60 °C for 2 min and 98 °C for 30 s; hold at 60 °C for 2 min. At the end of the reaction, chips were processed with QuantStudio™ 3D Digital PCR system (Applied Biosystems) and data analysis was performed using QuantStudio™ 3D Analysis Suite™ software (version 3.0.3).

### Variant calling and annotation from RNA seq data

Reads quality assessment was performed before the alignment step using FastQC v0.11.5 [http://www.bioinformatics.babraham.ac.uk/projects/fastqc/]. Sequencing reads were aligned to the GRCh38 genome assembly (with GENCODE v22 reference annotation) using a two-pass method with the STAR algorithm v2.7.3a [85]. Following the methods used by the International Cancer Genome Consortium (ICGC) [https://github.com/akahles/icgc_rnaseq_align], the two-pass method includes a splice junction detection step. Aligned reads in BAM format were sorted, and duplicate reads were flagged using Picard MarkDuplicates v2.9.0-1 [https://broadinstitute.github.io/picard/]. Systematic biases affecting the assignment of base quality scores by the sequencer were corrected using a BQSR procedure in a two-stage process performed by GATK tools (v4.1.4.1) BaseRecalibrator and ApplyBQSR [86]. Somatic short mutations (single-nucleotide variants and insertions/deletions) were called using GATK Mutect2 in tumor-only mode. Variants with sequencing depth >10 were retained and functionally annotated using ANNOVAR (with refGene, avsnp150, ljb26_all, cosmic90, clinvar_20190305, intervar_20180118, gnomad211_exome databases) [87].

### Cytokines assays

80-Cytokines analyses were conducted using a membrane-based antibody array, Cytokine Array C5 (RayBiotech Life, Inc., GA, USA) according to manufacturer’s protocol. Briefly, membranes were incubated with blocking buffer and then overnight incubated with 1 mL of neat medium under gentle rotation at 4 °C. Membranes were washed and cytokines were detected though HRP-Streptavidin/Biotin chemiluminescence detection using ChemiDoc MP Imaging System (Bio-Rad). Spot intensity was quantified using QuatityOne software (Bio-Rad) and data were then analyzed with the RayBio Antibody Array Analysis Tool (RayBiotech), which subtracted the background and the normalized data to the positive control signals.

### Extracellular vesicles isolation and phenotypical analysis

About 30 mL of exhausted media from co-cultures were intended to EV isolation. Media were initially cleared of any cell debris by centrifugation of 1000 *g* for 10 min then concentrated using through Centricon Plus-70 centrifugal filter devices (Merck Millipore). EV isolation was performed by size exclusion chromatography using qEV10 Size Exclusion Columns (70 nm, Izon Science) according to the manufacturer’s recommendations. After column equilibration and flushing with PBS 1X, we collected EV enriched 7–9 fractions using 0.5 mL per fraction and performed particle concentration of individual fractions by Nanoparticle Tracking Analysis (NTA). NTA analyses were performed using a NanoSight NS300 (Malvern Instruments, UK), equipped with NTA 2.3 analytical software laser, measurements were settled according to the manufacturer’s instructions diluting samples with 0.1 um filtered PBS1X. Since the EV quantifications indicated that the highest concentration was within fractions 7–8, we combined those fractions for subsequent experiments.

EVs phenotypical analyses were performed using MACSPlex Exosome kit human (Miltenyi Biotech) following the manufacturers protocol opting for overnight capture, suggested for the culture medium. This kit enables the detection of 37 markers (CD1c, CD2, CD3, CD4, CD8, CD9, CD11c, CD14, CD19, CD20, CD24, CD25, CD29, CD31, CD40, CD41b, CD42a, CD44, CD45, CD49e, CD56, CD62p, CD63, CD69, CD81, CD86, CD105, CD133.1, CD142, CD146, CD209, CD326, HLA- ABC, HLA-DR DP DQ, MCSP, ROR1 and SSEA-4) simultaneously, and two isotype controls (mIgG1 and REA control) corresponding to the antibodies. Samples staining was performed as in [88]. The samples were washed with MACSPlex buffer and analyzed on a BD FACS Canto equipped with two lasers, 488 nm, and 630 nm (Becton Dickinson), running a BD FACSDiva software (Becton Dickinson) recording a minimum of 50 events for each population of specific beads. 39 bead populations (37 exosomal surface epitopes + 2 isotype controls) were distinguished by different fluorescence intensities detected in the FITC, PE, and APC channels. Collected data were analysed by BD FACSDiva software (Becton Dickinson), median fluorescence intensity (MFI) was “background corrected” by blank samples and isotypes controls subtractions.

## Supplementary information

Normalized_Counts

Normalized_miRNA

SUPPLEMENTARY FIGURES

Movie 1

Supplemental Table S1

## Data Availability

All relevant data supporting the key findings of this study are available within the article and its Supplementary Information files or from the corresponding author upon reasonable request.
