# Supplementary material for: Gastroesophageal circulating tumor cell crosstalk with peripheral immune system guides CTC survival and proliferation
Source: Cell Death Dis. 2025 Mar 29;16(1):223. doi: 10.1038/s41419-025-07530-2 (PMC11954855; doi:10.1038/s41419-025-07530-2)

**SUPPLEMENTARY FIGURES**

**Supplementary Figure S1. Heat map of the top-50 differentially expressed genes between CTC-derived cell lines and TCGA ESCA and STAD datasets.** Rows: genes; Columns: CTC samples (yellow) and TCGA samples (gray); red color represents the expression above the average across samples, blue color represents the expression below the average across samples.

**Supplementary Figure S2. EV concentration in media secreted by different co-cultures.** EVs previously enriched by size exclusion chromatography (fraction 7 blue, fraction 8 orange) were analyzed using nanoparticle tracking analysis (NTA) with NanoSight NS300 Instrument.

**Supplementary Figure S3. Network analysis on the targets of the CTC cell line EV-specific miRNAs.** Network enrichment analysis conducted on the targets of CTC EV enriched miRNAs (n=18) obtained using Metacore.

**Supplementary Figure S4. Representative immunofluorescence staining of differentiated leukocytes in the 2D-compartment** indicating differentiated monocytes CD11c+/CD14+ with a macrophages M2-like profile (CD206+/CD163+) and positive for HLA-DR, while negative for dendritic markers CD1a, CD80, CD83. CD80 (yellow), CD206 (green), CD163 (red), CD11c (yellow), CD14 (green), HLA-DR (red), CD83 (yellow), CD1a (red), nuclei are in blue.

**Supplementary Figure S4. Cytokine profile of CTC co-cultures.** Human Cytokine Antibody Array Kit was used to measure the presence of 80 cytokines in the supernatant of different co-cultures. (a) Array cytokines map, colored squares indicate distinctive cytokines detected in supernatants: unconditioned medium (yellow), 3D-compartment cells (green), co-culture (red). (b-c) Cytokine Array membranes of supernatant of medium (b) co-colture compartments (c). (d) Quantitative analysis of the distinctive cytokines detected by arrays. Pixel density was determined by measuring spot chemiluminescent signals in Quantity One software; Raybiotech tool was used to calculate normalized signal intensity using pixel density adjusted with background subtraction and internal positive controls. (e) Cytokine Array of supernants of co-cultures from CTC-success co-culture (n=3) (RGCTC, NM9CTC, CACTC) and CTC-failure co-culture (n=2) (FSCTC, VICTC). (f) Quantitative analysis of the distinctive cytokines detected by arrays of supernants of co-cultures from CTC-success co-culture (n=3) (RGCTC, NM9CTC, CACTC) and CTC-failure co-culture (n=2) (FSCTC, VICTC).

**Supplementary Figure S5. Membrane epitopes profiles of EVs secreted in the co-cultures.** EV samples were analyzed by MACSPlex Exosome Kit surface marker antibody panel and flow cytometry. (a) Phenotypic profiles of EVs from healthy donor PBMC co-culture and CTC-success co-culture without proliferating CTC. HLA-DR, CD11C, CD40 and CD29 were detected only EVs of healthy donor PBMC co-culture (highlighted in yellow) (MFI > 500). In contrast, the membrane integrin CD41b was distinctive of EVs of CTC-success co-culture (highlighted in green) (MFI >500). (b) Comparison of phenotypic profiles of EVs in CTC-success co-culture with (w) (checkered pattern) and without (w/o) (plain colors) proliferating CTCs. CD326 (EpCAM) was detected only in co-cultures with CTCs (highlighted in pink), CD11c and MCSP1 were detected only in co-cultures without CTCs (highlighted in cyano) (MFI>100).

**Supplementary Figure S6 Principal component analysis (PCA) of EV-specific miRNAs EVs from distinct in-vitro cell cultures.** Comparison of EV-specific miRNAs from supernants of healthy donor co-culture (N=3) (blue spheres), CTC-failure co-culture (N=2) (green triangles), CTC-success co-culture, with growing CTCs (N=2) (gray) and without CTCs (N=2) (orange), CTC-derived cell lines expanded after the co-culture (N=4) (red) and commercial cell lines (N=2) (bronze).

**Supplementary Figure S7 Heatmap and cluster dendrogram of the 5 miRNA contained only in EVs of the co-cultures with featured proliferating CTC.**

**Supplementary Figure S8 Network analysis on the targets of the EV-specific miRNAs secreted by CTC-success co-culture.** Network enrichment analysis conducted on the targets of CTC-success co-culture EV enriched miRNAs (n=5) obtained using Metacore

**Suplementary Figure S10. Illustration of the co-culture systems**. Schematic illustration of PBMC and CTC enrichment steps starting from peripheral blood sample and cell seeding of co-culture compartments. Briefly, PBMCs were isolated by density gradient centrifugation and seeded on the top of the 3D scaffold of the well insert. CTCs were enriched using a negative depletion method and seeded into suspension. Co-cultures were maintained in serum-free medium in a humidified incubator at 37°C with 4% O_2_ and 5% CO_2_

**MOVIE LEGEND**

**Movie: Timelapse imaging of tail region of a living zebrafish embryo injected with CTCs**. 24-39h after injection intravascular CTCs (red) circulate passively and actively through interactions with endothelium (green). Image acquisition was performed with 20X magnification Zoom 2X MaxIP with Nikon Eclipse Ti2 confocal microscope using NIS Elements software.


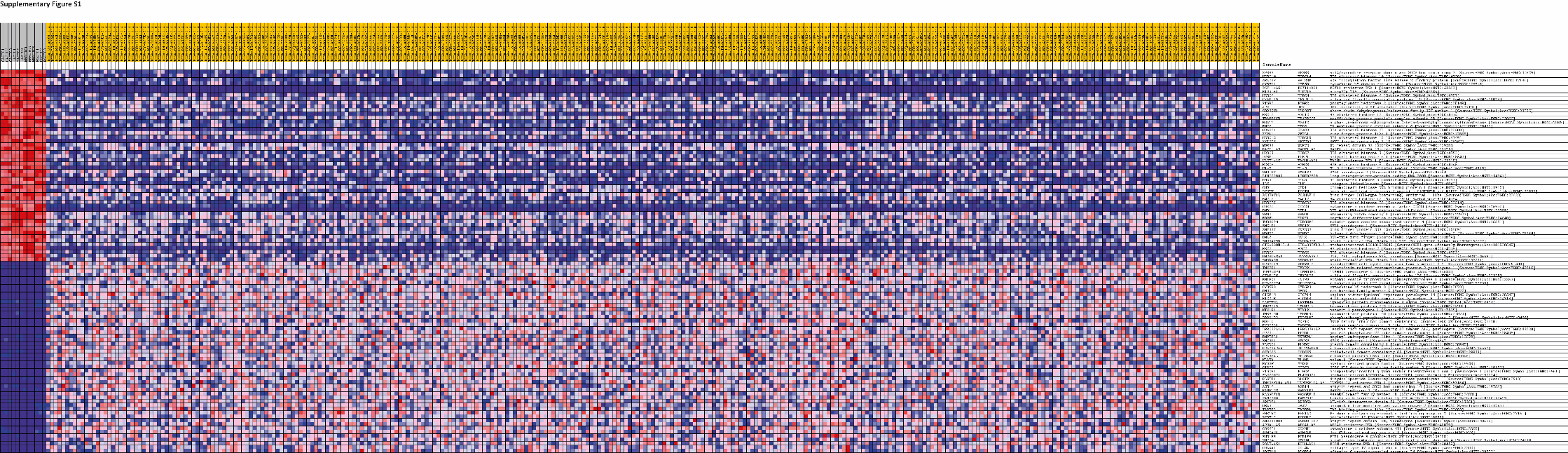


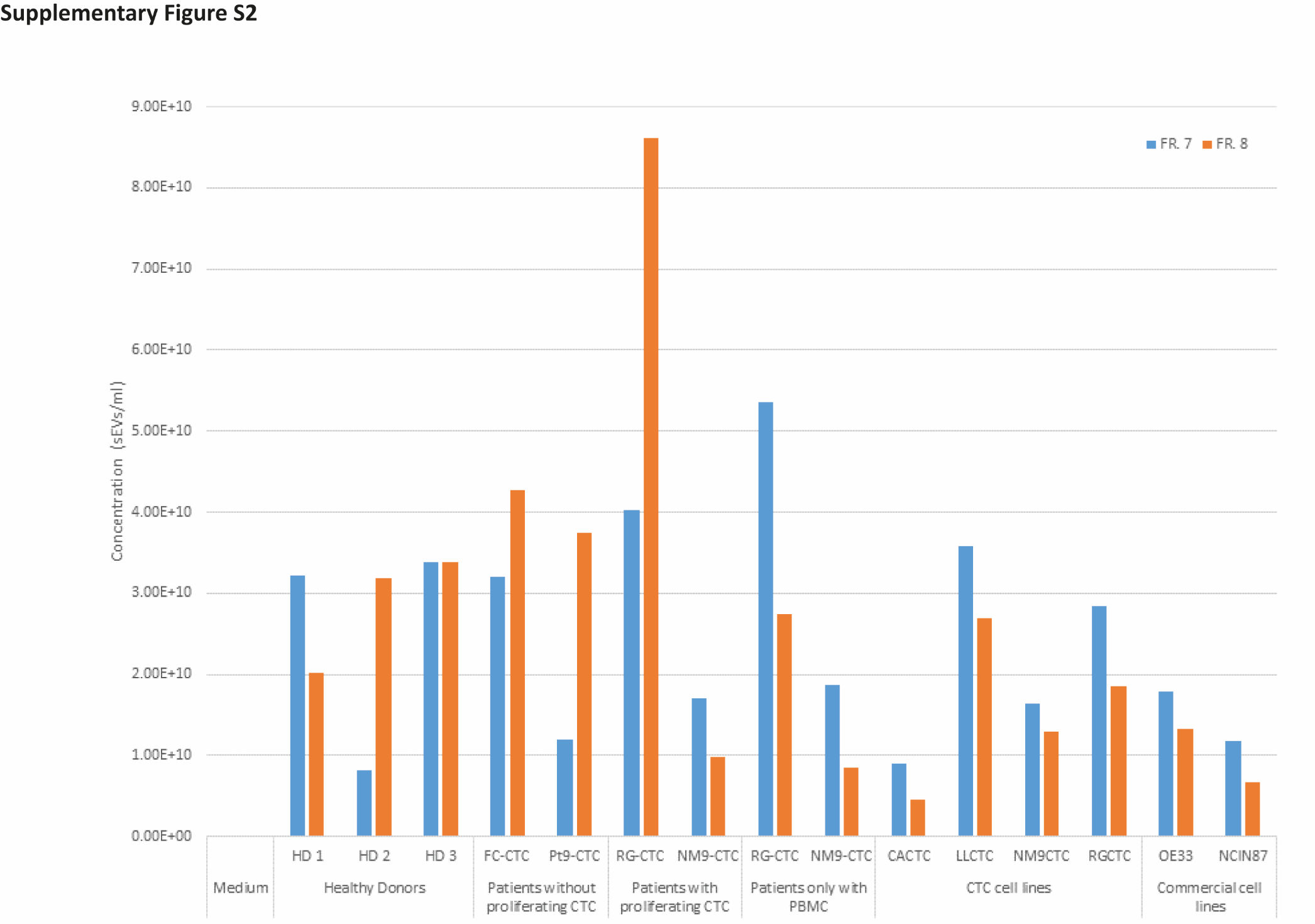


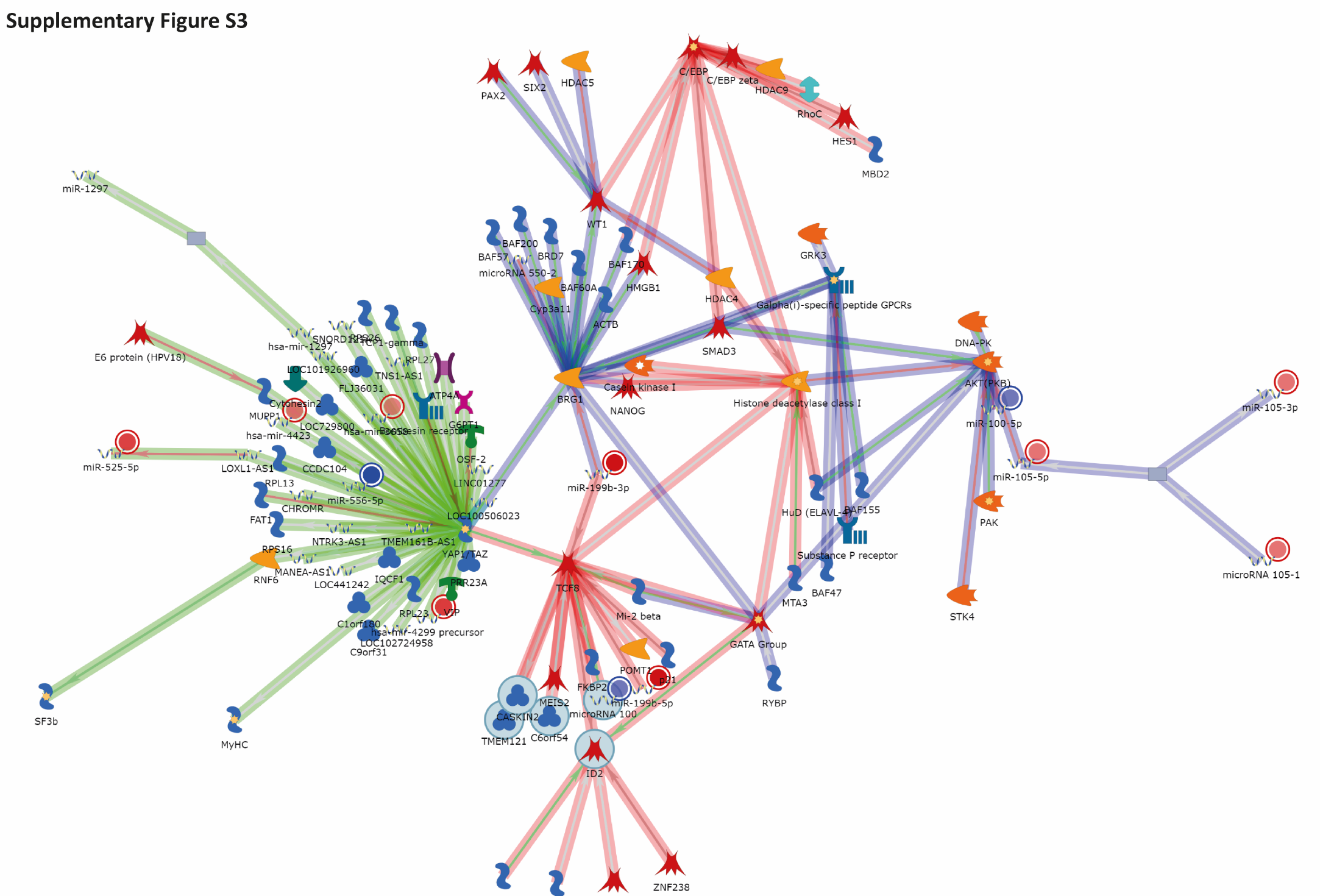


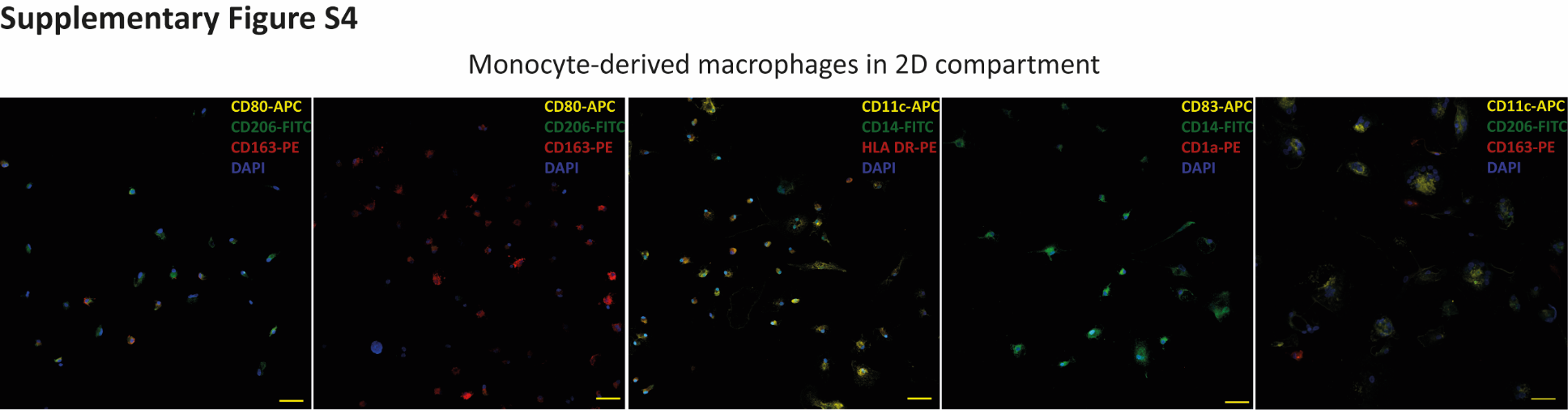


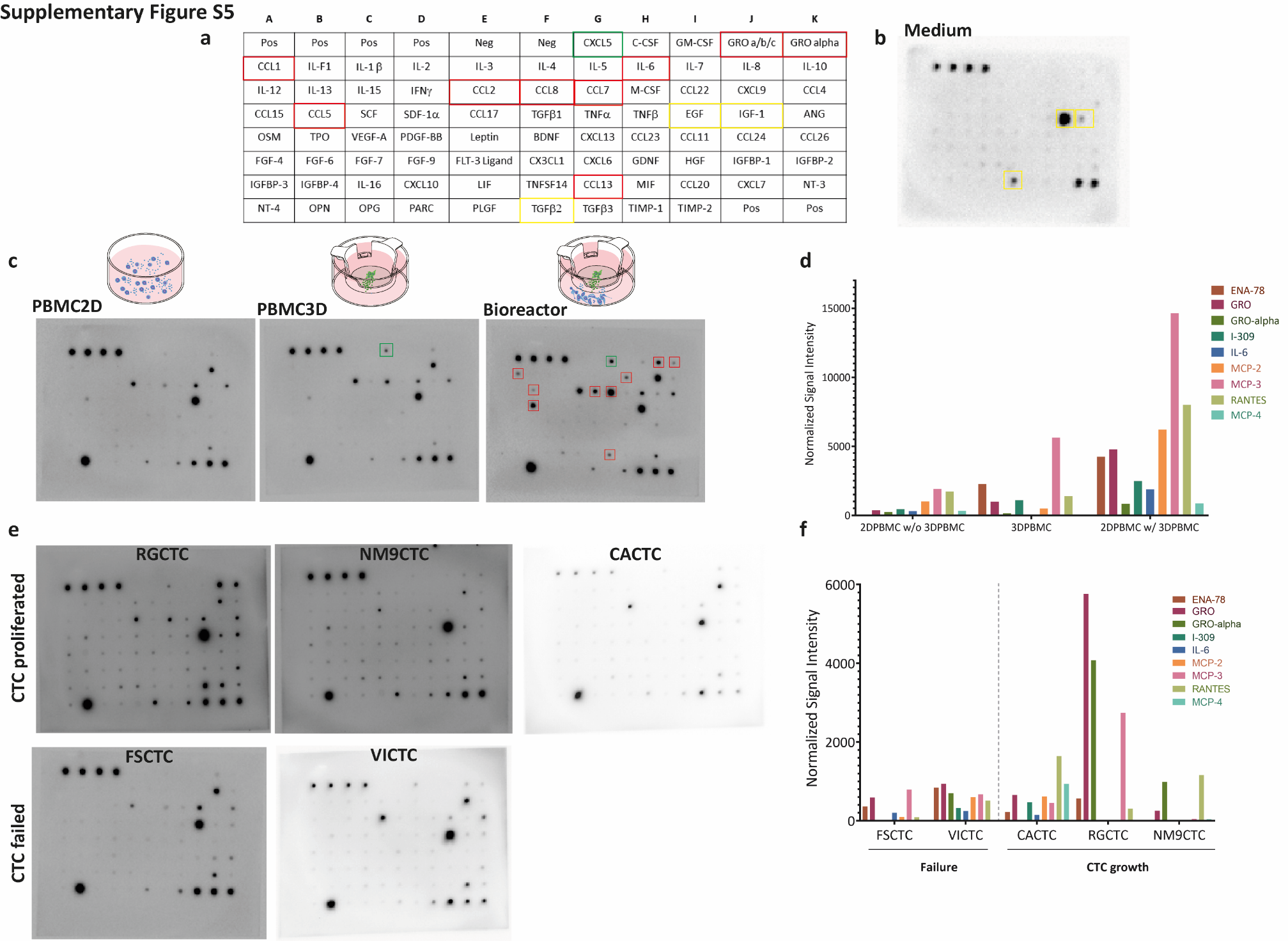


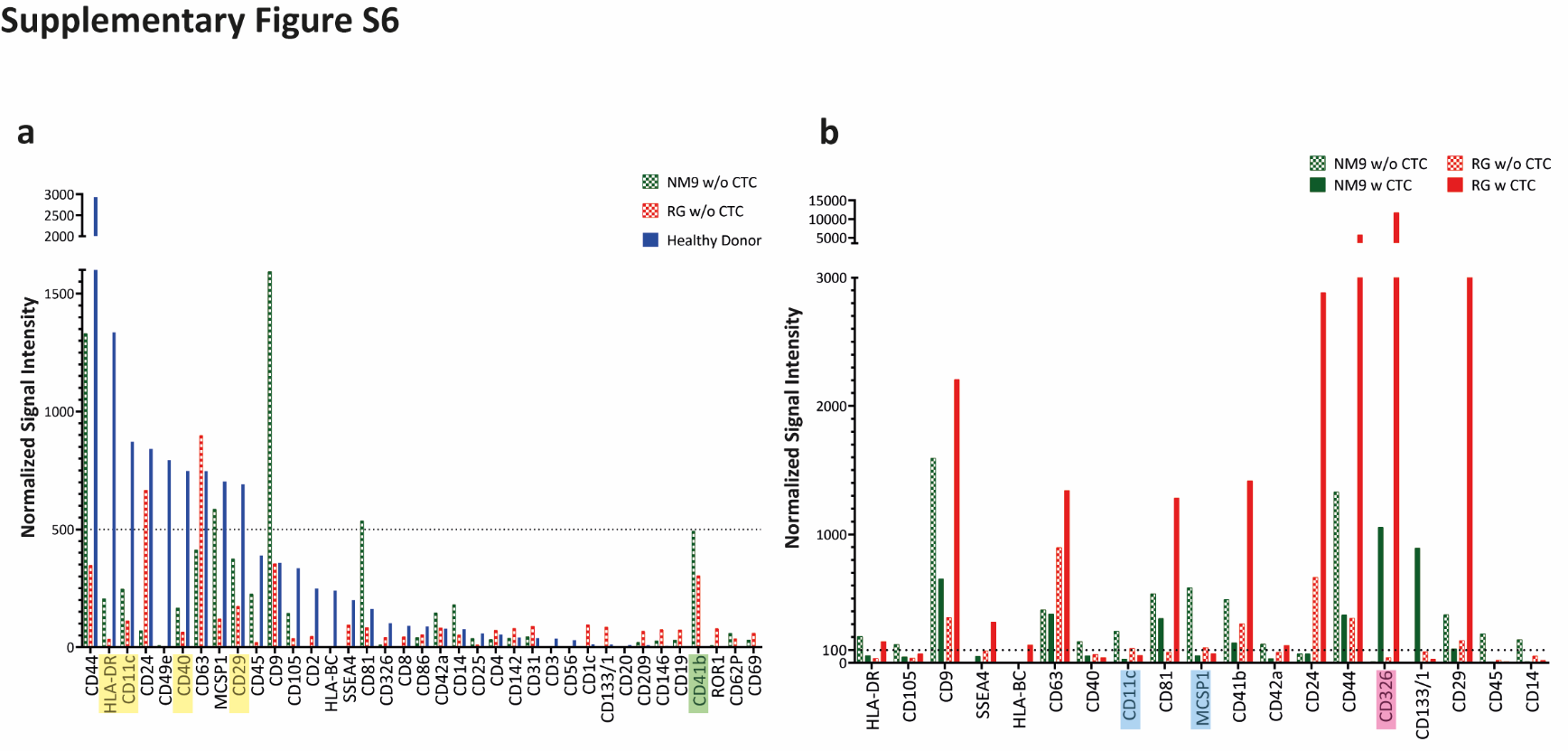


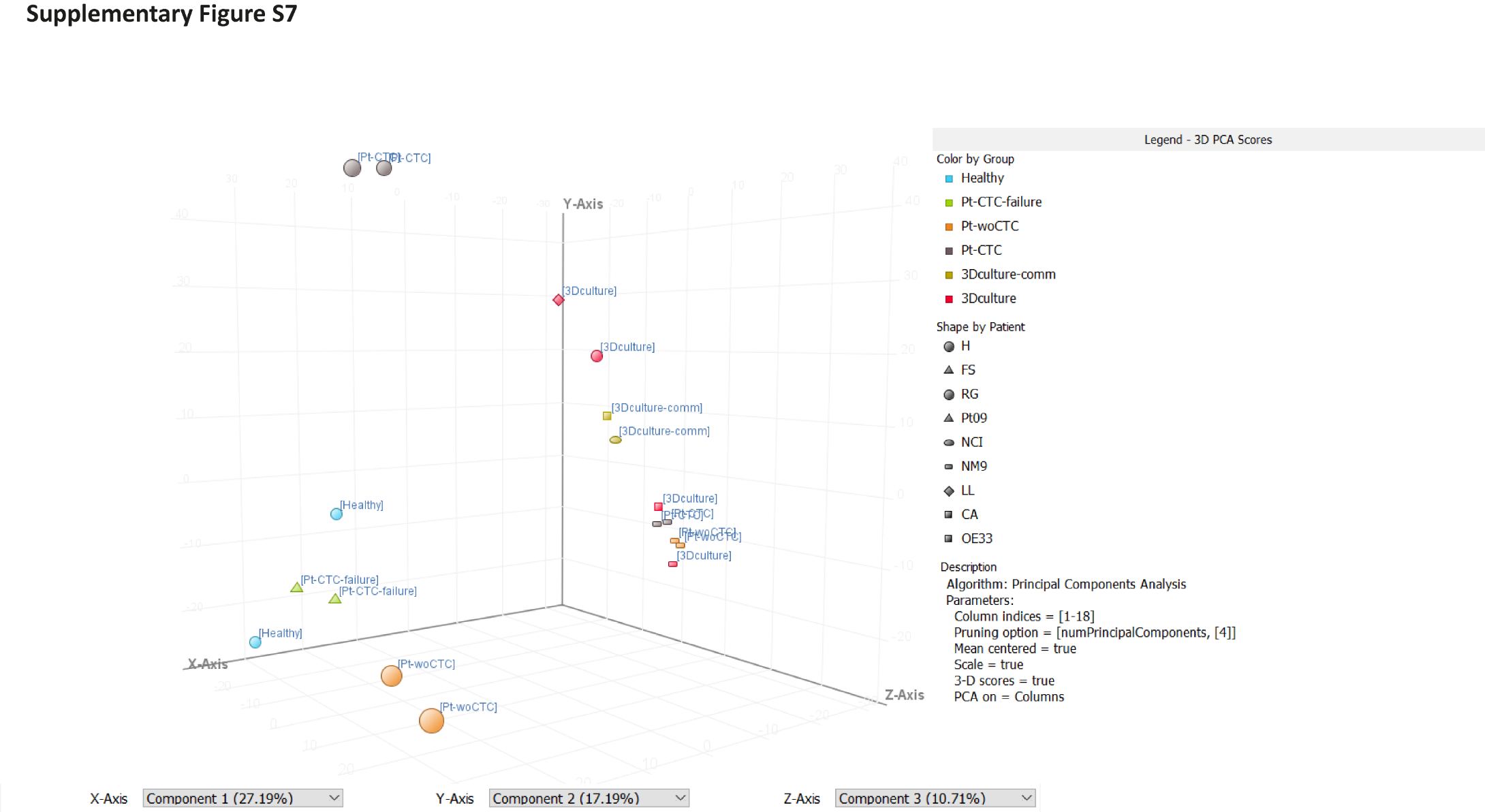


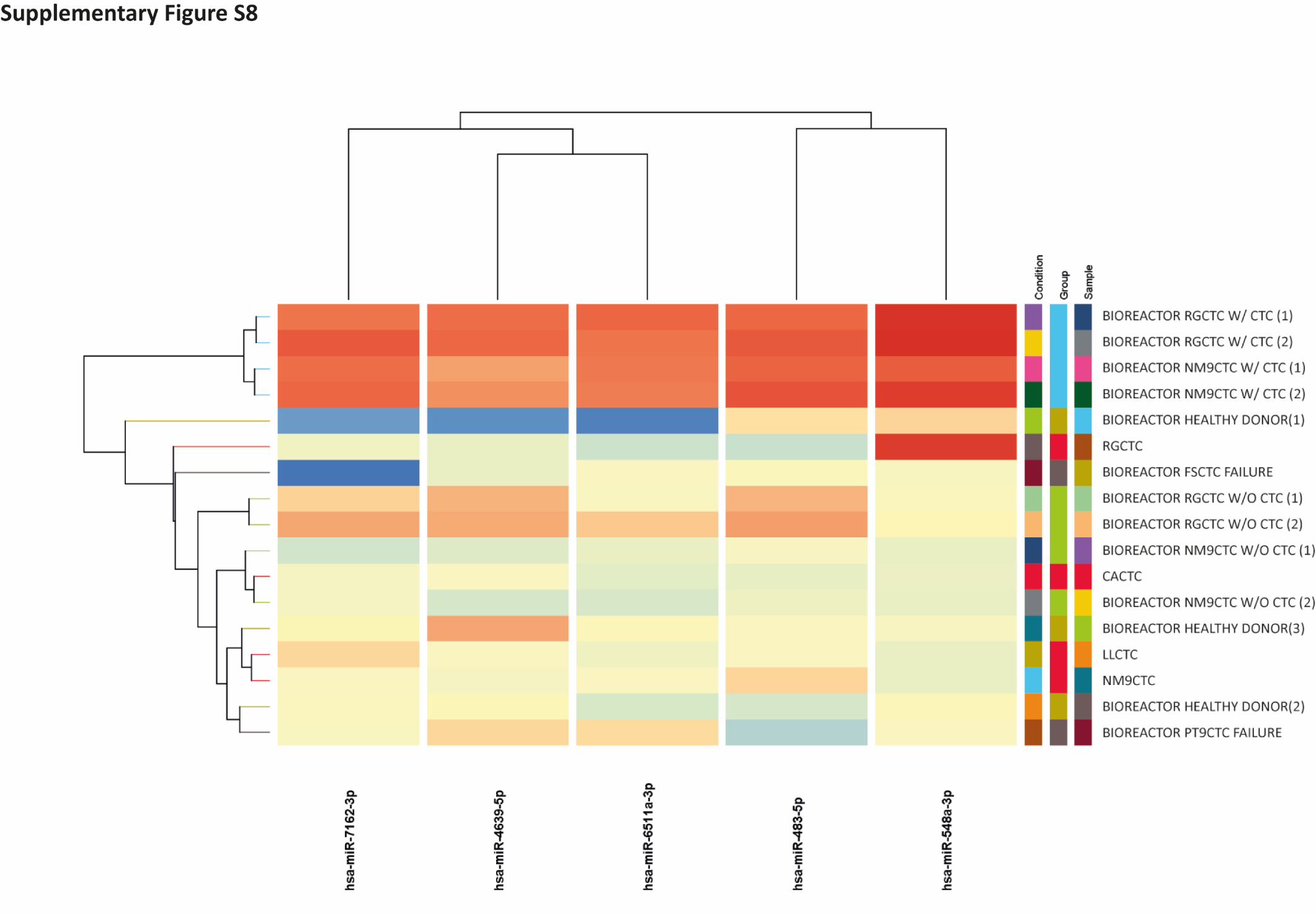

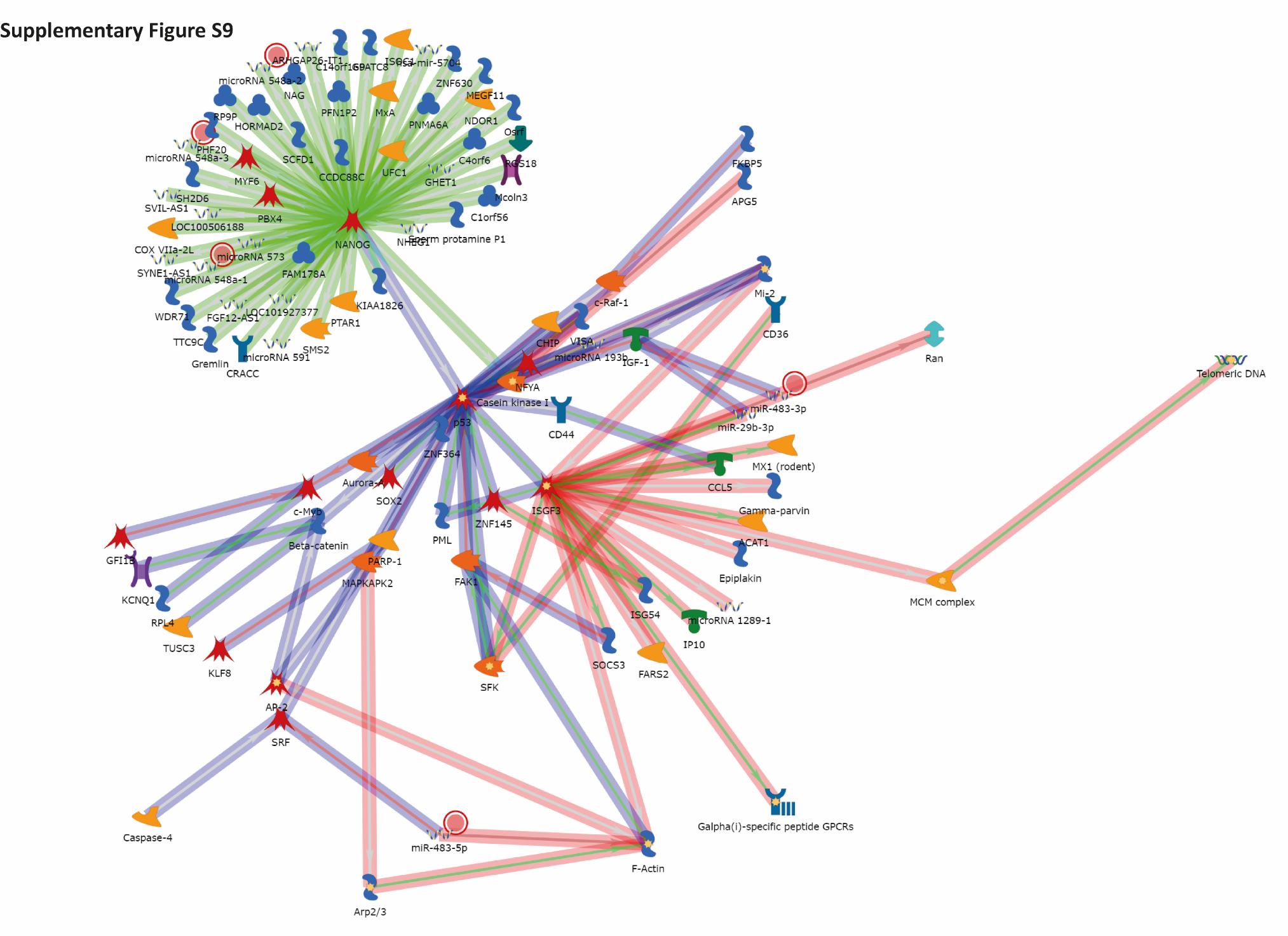


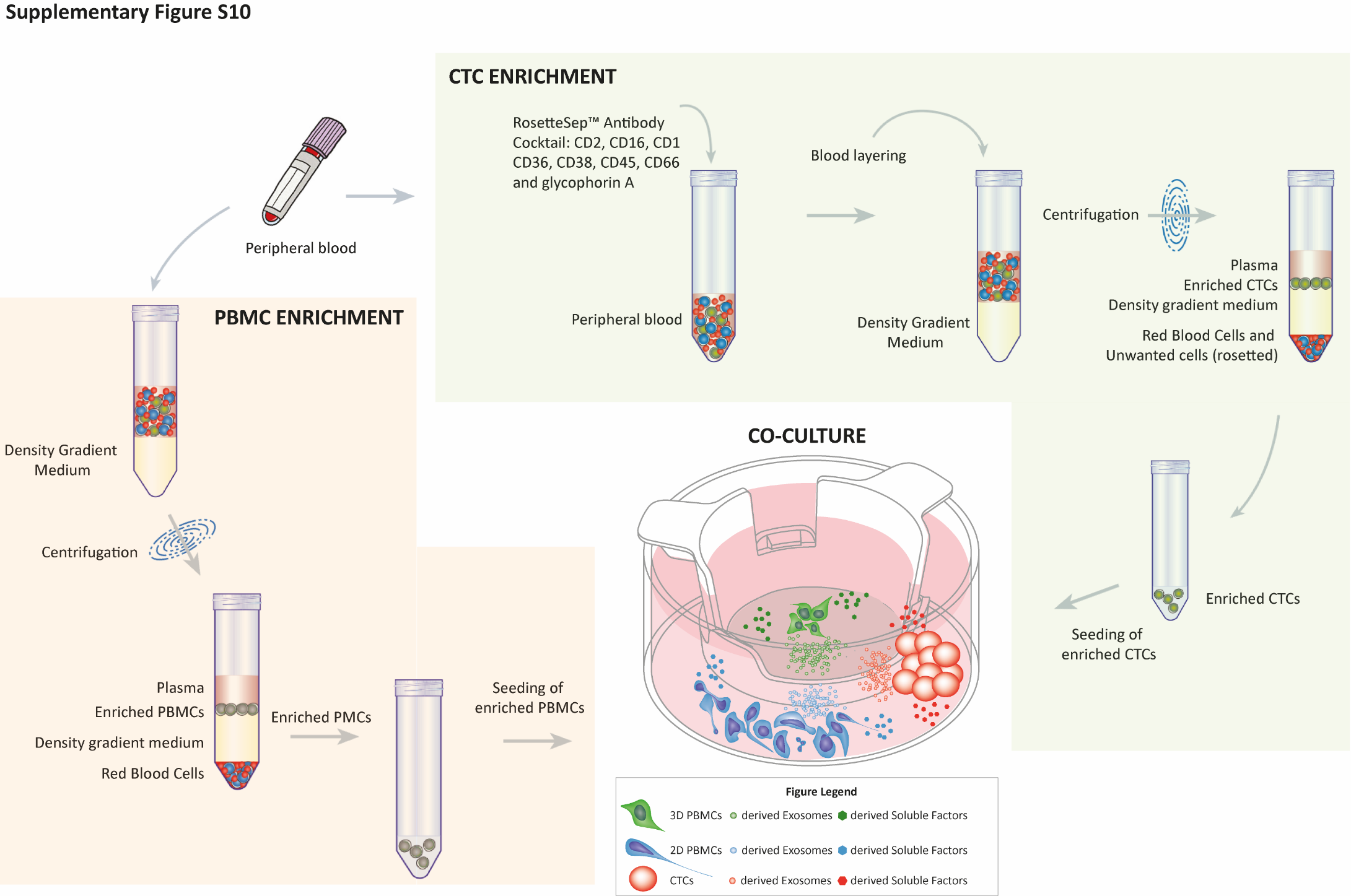

Supplement: Supplementary file 3 — SUPPLEMENTARY FIGURES [file 41419_2025_7530_MOESM3_ESM.docx]
